# Acquisition and loss of virulence-associated factors during genome evolution and speciation in three clades of *Bordetella* species

**DOI:** 10.1186/s12864-016-3112-5

**Published:** 2016-09-30

**Authors:** Bodo Linz, Yury V. Ivanov, Andrew Preston, Lauren Brinkac, Julian Parkhill, Maria Kim, Simon R. Harris, Laura L. Goodfield, Norman K. Fry, Andrew R. Gorringe, Tracy L. Nicholson, Karen B. Register, Liliana Losada, Eric T. Harvill

**Affiliations:** 1 Department of Veterinary and Biomedical Sciences, Pennsylvania State University, University Park, PA 16802 USA; 2 The Millner Centre for Evolution and Department of Biology and Biochemistry, University of Bath, Bath, UK; 3 J. Craig Venter Institute, Rockville, MD USA; 4 Pathogen Genomics, The Wellcome Trust Sanger Institute, Wellcome Trust Genome Campus, Hinxton, Cambridge, UK; 5 Public Health England, Respiratory and Vaccine Preventable Bacteria Reference Unit, London, UK; 6 Public Health England, Porton Down, Salisbury, UK; 7 USDA, Agricultural Research Service, National Animal Disease Center, Ames, IA USA; 8 Singapore Centre on Environmental Life Sciences Engineering, Lee Kong Chian School of Medicine, Nanyang Technological University, Singapore 637551, Singapore; 9 Department of Infectious Diseases, College of Veterinary Medicine, University of Georgia, Athens, GA 30602 USA

**Keywords:** Bordetella, Evolution, Virulence factor, Gene acquisition, Gene loss

## Abstract

**Background:**

The genus *Bordetella* consists of nine species that include important respiratory pathogens such as the ‘classical’ species *B. bronchiseptica*, *B. pertussis* and *B. parapertussis* and six more distantly related and less extensively studied species. Here we analyze sequence diversity and gene content of 128 genome sequences from all nine species with focus on the evolution of virulence-associated factors.

**Results:**

Both genome-wide sequence-based and gene content-based phylogenetic trees divide the genus into three species clades. The phylogenies are congruent between species suggesting genus-wide co-evolution of sequence diversity and gene content, but less correlated within species, mainly because of strain-specific presence of many different prophages. We compared the genomes with focus on virulence-associated genes and identified multiple clade-specific, species-specific and strain-specific events of gene acquisition and gene loss, including genes encoding O-antigens, protein secretion systems and bacterial toxins. Gene loss was more frequent than gene gain throughout the evolution, and loss of hundreds of genes was associated with the origin of several species, including the recently evolved human-restricted *B. pertussis* and *B. holmesii*, *B. parapertussis* and the avian pathogen *B. avium*.

**Conclusions:**

Acquisition and loss of multiple genes drive the evolution and speciation in the genus *Bordetella*, including large scale gene loss associated with the origin of several species. Recent loss and functional inactivation of genes, including those encoding pertussis vaccine components and bacterial toxins, in individual strains emphasize ongoing evolution.

**Electronic supplementary material:**

The online version of this article (doi:10.1186/s12864-016-3112-5) contains supplementary material, which is available to authorized users.

## Background

The genus *Bordetella* belongs to the class *Betaproteobacteria* and contains nine species that include the so-called ‘classical’ bordetellae consisting of *B. pertussis*, *B. parapertussis* and *B. bronchiseptica*. The highly infectious human pathogen *B. pertussis* is the causative agent of whooping cough, a respiratory disease that is particularly serious and sometimes fatal in infants and in elderly people. One lineage of *B. parapertussis* causes pneumonia in sheep, while the other lineage causes a whooping cough-like disease in children [1]. *B. bronchiseptica*, a respiratory pathogen of diverse mammals, causes a variety of pathologies ranging from chronic and often asymptomatic infection to more acute diseases such as kennel cough in dogs, bronchitis in cats, and bronchopneumonia and atrophic rhinitis in pigs [2–4].

Multi-locus Sequence Typing (MLST) of the genus *Bordetella,* supported by targeted genome sequencing revealed that *B. pertussis* and *B. parapertussis* independently evolved from different lineages of a *B. bronchiseptica*-like ancestor [5–7]. Specifically, *B. parapertussis* likely evolved from a *B. bronchiseptica* ancestor within the Multi Locus Sequence Type (MLST) complex I, while strains of *B. bronchiseptica* complex IV and *B. pertussis* appear to share a common ancestor that branched off from complex I *B. bronchiseptica* [6]. Despite differences in host range and disease, the classical bordetellae are very closely related and share many important virulence factors, including putative adhesins such as pertactin (PRN), filamentous hemagglutinin (FHA) and fimbriae, and toxins such as adenylate cyclase toxin (ACT), pertussis toxin (PT) and dermonecrotic toxin (DNT) [5]. However, some virulence-associated factors are species-specific such as a recently identified type-VI secretion system (T6SS) in *B. bronchiseptica* [8] which is missing in *B. pertussis* and probably not functional in *B. parapertussis* due to missing subsets of genes and/or pseudogenes within this locus [7].

In addition to the classical bordetellae, the genus contains six other species that are phylogenetically distinct and are collectively referred to as ‘non-classical’: *B. hinzii*, *B. holmesii*, *B. avium*, *B. trematum*, *B. petrii* and *B. ansorpii* (Table 1). *B. holmesii* has been isolated with increasing frequency from outbreaks of pertussis-like illness, occasionally in conjunction with *B. pertussis* infections [9–11], and also causes septicemia and endocarditis in immunocompromised patients [12, 13]. Recent analysis of clinical *B. holmesii* genome sequences suggest that these strains are similar to *B. pertussis* in that all *B. holmesii* isolates belong to the same multilocus sequence type [14], however these clinical strains lack the important virulence genes that are encoded by *B. pertussis* [14, 15].

**Table 1:** Host specificity and disease caused by nine *Bordetella* species

*Bordetella* species	Number of analyzed genomes	Genome size (reference genome)	Host	Disease
*B. bronchiseptica*	58	5,338,400 bp	variety of mammals, including pigs, dogs, cats, seals, rabbits, horses, mice, sheep, humans	Broad variety of respiratory disease, from clinically unapparent to fatal pneumonia, such as snuffles in rabbits, atrophic rhinitis in pigs and kennel cough in dogs
*B. parapertussis*	2	4,773,551 bp	humans, sheep	Respiratory infection, Whooping Cough-like disease
*B. pertussis*	34	4,086,186 bp	humans	Respiratory infection, Whooping Cough
*B. hinzii*	6	4,885,897 bp	turkeys, rabbits, immunocompromised humans	Respiratory disease in turkeys and rabbits, septicemia in humans
*B. holmesii*	18	3,699,674 bp	humans	Respiratory infection, Whooping Cough-like disease, bacteremia
*B. avium*	1	3,732,255 bp	birds	Respiratory disease
*B. trematum*	4	4,485,537 bp	humans	Ear infection, wound infection
*B. ansorpii*	2	6,210,412 bp	humans	Epidermal cyst, wound infection
*B. petrii*	3	5,287,950 bp	humans, environmental	Wound infection (osteomyelitis), ear infection (chronic mastoiditis), chronic bronchorrhea, environmental

*B. hinzii* colonizes the respiratory tracts of poultry and was shown to cause disease during experimental infection in turkeys although the severity depended on the bacterial strain used for infection [16, 17]. In addition, *B. hinzii* has also been isolated from rodents [18, 19] and from immunocompromised humans with respiratory disease, septicemia and cholangitis [20–22].

*B. avium* has been isolated from the respiratory tract of turkeys, chickens and other poultry and causes a respiratory disease with the clinical symptoms known as coryza or bordetellosis in turkey chicks [23]. *B. avium* also infects other wild and domesticated birds, but domesticated turkeys are particularly susceptible [24]. More than half of the over 5,000 genes predicted in the genome of *B. bronchiseptica* strain RB50 have no orthologues in the much smaller genome of *B. avium* strain 197N, including genes encoding different O-antigen and capsular biosynthesis genes and important toxins of the classical bordetellae such as PT and ACT [25]. Likewise, nearly one third of the 3,417 predicted genes of *B. avium* do not have orthologues in *B. bronchiseptica*, including genes encoding unique hemagglutinins, a variety of fimbriae, O-antigen biosynthesis proteins and capsular biosynthesis proteins [25].

Contrary to many other bordetellae, *B. trematum* has never been found in the respiratory tract. Instead, it has been isolated from wound infection after severe burns, from wound infection resulting from a diabetic ulcer and from chronic otitis media in humans [26–28] as well as from the rumen of cattle in Korea [29].

*B. ansorpii* is the species with the fewest known cases, it has been documented just twice. It has been isolated from a purulent epidermal cyst of a chemotherapy patient in Korea [30] and from a catheter of a chemotherapy patient in the UK [31], suggesting that *B. ansorpii* may be an opportunistic pathogen of immunocompromised humans.

The most adaptable of the non-classical species seems to be *B. petrii*. This species was originally described as environmental because it was first isolated from an anaerobic bioreactor culture enriched from river sediment and demonstrates remarkable metabolic versatility [32]. The genome sequence revealed that *B. petrii* is missing toxins of the classical bordetellae such as PT, ACT and DNT [33], although it retains both the master virulence regulator of the pathogenic *Bordetella* species, BvgAS, as well as FHA. The combination of a broad metabolic versatility and virulence traits associated with the classical bordetellae suggested that *B. petrii* represents an intermediate between free-living environmental bacteria and obligate bacterial pathogens [33]. Supporting this view, other isolates have been obtained from clinical cases of bone infection [34], ear infection [35], and from sputum of a patient with chronic bronchorrhea symptoms [36], indicating that these strains are able to colonize and cause disease in human hosts.

Recent efforts have generated numerous additional genome sequences from many *Bordetella* species, including *B. bronchiseptica* isolated from 11 different hosts [37] and *B. pertussis* outbreak strains [38–40]. Of particular interest are recently sequenced but only partially characterized genomes of non-classical *Bordetella* species, including sequences of *B. holmesii* [14, 15], *B. hinzii* [41, 42] and *B. trematum* [29, 41]. Here we analyze the evolution of the genus *Bordetella* based on genome sequences from all nine species, including novel draft genomes from *B. ansorpii* and *B. trematum*. Based on phylogenetic trees created from a genome-wide sequence comparison and a gene content analysis, we show that the bordetellae evolved into three distinct clades of species that consist of: clade A) the three classical bordetellae *B. bronchiseptica*, *B. pertussis* and *B. parapertussis*, clade B) the non-classical species *B. avium*, *B. hinzii*, *B. holmesii* and *B. trematum*, and clade C) the opportunistic pathogens *B. petrii* and *B. ansorpii*. The sequence-based and gene content-based phylogenies were largely congruent, suggesting that sequence and gene content co-evolved despite loss of hundreds of genes that was associated with the origin of several species, including the recently evolved human-restricted *B. pertussis* and *B. holmesii*, *B. parapertussis* and the avian pathogen *B. avium*. By mapping presence and absence of virulence-associated genes and gene clusters to the phylogenetic tree, we show that numerous events of lineage-specific, species-specific and isolate-specific gene acquisition and gene loss shaped the evolution and speciation in the genus whereby gene loss was more frequent than gene gain throughout the evolution.

## Results

### Data set

Selecting strains for greatest diversity by various measures, we sequenced and analyzed a dataset of 128 genomes from all nine known *Bordetella* species, including 94 genomes from the classical species *B. bronchiseptica*, *B. parapertussis* and *B. pertussis*, and 34 genomes from the non-classical *Bordetella* species *B. holmesii*, *B. hinzii*, *B. avium*, *B. trematum*, *B. petrii* and *B. ansorpii*. Thirty-seven of the 58 *B. bronchiseptica* strains belong to MLST complex I while 21 belong to complex IV. *B. bronchiseptica* isolates originated from humans (17 strains), a variety of different mammals (31 strains), turkeys (9 strains), and from an unknown host (1 strain; Additional file 1: Table S1) [5, 7, 37, 43, 44]. The data set further contains the genomes of 34 *B. pertussis* strains from human hosts [5, 7, 38, 45] and the genomes of *B. parapertussis* strain 12822 from a human (*B. parapertussis*_hu_) [5] and strain Bpp5 from a sheep (*B. parapertussis*_ov_) [7]. The non-classical bordetellae include 18 genomes of the emerging human respiratory pathogen *B. holmesii* [14, 15], 6 genomes of the respiratory pathogen *B. hinzii* [42] from turkeys (3 strains), human (2 strains) and rabbit (1 strain), one genome of the avian respiratory pathogen *B. avium* [25], 4 genomes of *B. trematum* from wound and ear infection in humans ([41] and this study), 3 genomes from environmental *B. petrii* isolates [33], and genomes of the only 2 described isolates of *B. ansorpii* from human wound infection (Table 1, Additional file 1: Table S1).

### Core genome based on genome wide SNPs

We generated a sequence alignment of all 128 genomes using the genome of *B. bronchiseptica* strain RB50 [5] as a reference. The other genome sequences were each cut into overlapping DNA fragments of 54 bp in length and aligned against the reference genome in order to generate a genome-wide, multiple sequence alignment of 5,339,179 bp in length. Only 184,916 bp of sequence which contains fragments of 827 genes was shared among all 128 analyzed genomes, representing the core genome of the *Bordetella* genus (Table 2). The remaining sequence was either absent in at least one of the genomes or was so divergent that it did not align. For example, an average of 1,272,049 bp from each of the *B. hinzii* genomes aligned against the genome of *B. bronchiseptica* RB50, of which 1,202,277 bp (4,071 polymorphic sites; 0.34 %) were shared between all six *B. hinzii* genomes (Table 2). The 34 genomes of all non-classical *Bordetella* species were so diverse that they shared only 214,564 bp of which 47,646 (22.2 %) were polymorphic. In contrast, 2,569,320 bp of sequence (3.37 % polymorphic) were present in all 94 genomes of the classical bordetellae (Table 2). We note that the size of the core genome for each species and the number of polymorphic nucleotides within each species may be affected by the number of genome sequences per species. These data reveal substantial sequence conservation within species but considerably more sequence divergence as well as gene gain and loss between species.

**Table 2:** Number of core genes, number of shared nucleotides and *R* ^2^ correlation between sequence and gene content evolution

Data set	Core genome: Gene content	Core Genome: Shared nucleotide loci (bp)			Correlation between sequence and gene content evolution	
		Total	Polymorphic sites		*R* ^2^	*P* value
		bp	bp	%		
128 bordetellae	850	184,916	42,577	23.025	0.74	0.00001
94 classical bordetellae	1828	2,569,320	86,508	3.367	0.29	0.00001
58 *B. bronchiseptica*	3284	3,908,069	121,219	3.102	0.37	0.00001
2 *B. parapertussis*	3592	4,249,275	19,301	0.454	NA	NA
34 *B. pertussis*	2632	3,265,138	2,543	0.078	0.68	0.00001
34 non-classical bordetellae	1209	214,564	47,646	22.206	0.81	0.00001
18 *B. holmesii*	2654	702,836	144	0.021	0.09	0.04148
6 *B. hinzii*	3978	1,202,277	4,071	0.339	0.21	0.03026
4 *B. trematum*	3629	948,918	2,394	0.252	0.63	0.04192
1 *B. avium*	NA	NA	NA	NA	NA	NA
2 *B. ansorpii*	4033	1,048,733	29,556	2.818	NA	NA
3 *B. petrii*	2629	741,871	91,356	12.314	0.74	0.16443

*NA* not applicable

### Phylogeny based on genome wide SNPs

**Fig. 1:**
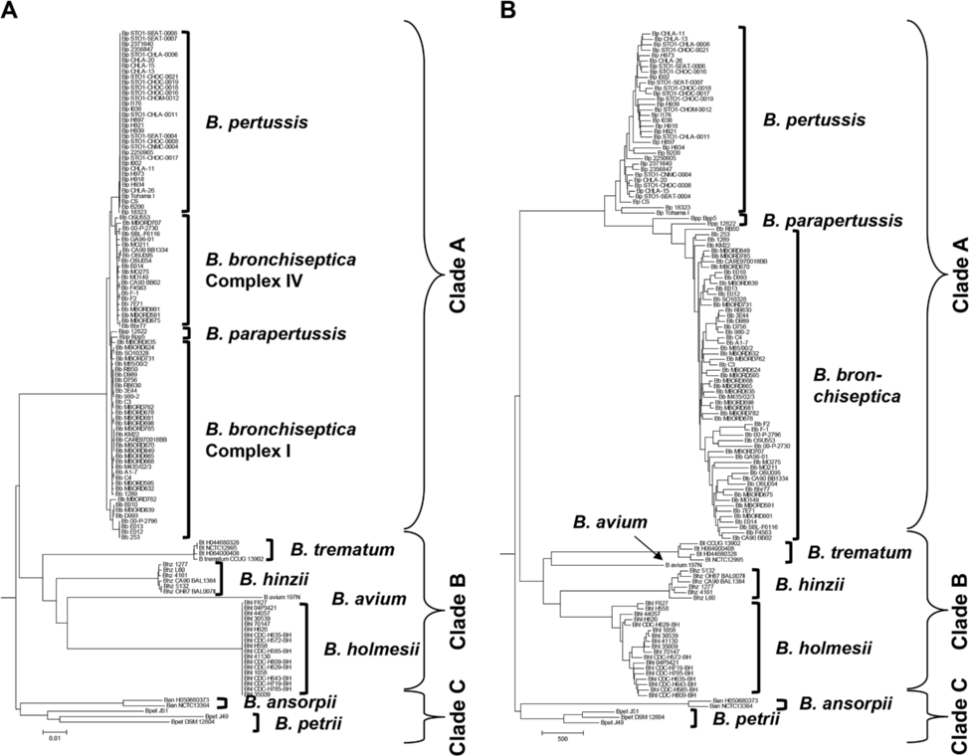
Phylogenetic structure (Neighbor-joining trees) according to **a** a genome-wide sequence alignment, and **b** presence or absence of genes in 128 genomes from nine species of the genus *Bordetella*. The bordetellae evolved into 3 clades of species: the phylogenetically oldest clade C formed by *B. petrii* and *B. ansorpii*, clade B formed by *B. trematum*, *B. hinzii*, *B. avium* and *B. holmesii*, and clade A containing the classical bordetellae *B. bronchiseptica*, *B. parapertussis* and *B. pertussis*. The Neighbor-joining trees were rooted based on tree topologies of 16S rRNA gene and 23S rRNA gene sequence and ATP-synthase protein phylogenies (Additional file 1: Figure S1)

In order to understand how the *Bordetella* species are related to each other, we generated a phylogenetic tree that is based on the 184,916 bp core genome which contains 42,577 polymorphic sites (Fig. 1a). To root the core genome-based tree, we analyzed the 16SrRNA gene and 23SrRNA gene phylogenies of the genus as well as the phylogeny based on the concatenated protein sequences of eight ATP synthase genes (2,143 amino acids, 293 polymorphic among the *Bordetella* species) and rooted these trees with the corresponding sequences of the related *Betaproteobacteria Ralstonia solanacearum* and *Burkholderia pseudomallei* (Additional file 1: Figure S1). In all three phylogenetic trees, *B. ansorpii* and *B. petrii* were located closer to the root than any other *Bordetella* species. Based on this information, we rooted the core genome-based tree (Fig. 1a).

The sequence-based tree of *Bordetella* species revealed three distinct clades. Clade C, formed by *B. petrii* and *B. ansorpii,* was located closest to the root of the tree of all *Bordetella* species and thus likely branched off first during the evolution of the genus (Fig. 1a). Subsequently, the ancestors of the remaining species split into two additional clades. Clade B consisted of *B. holmesii*, *B. hinzii*, *B. avium* and *B. trematum* and clade A contained the classical bordetellae, *B. bronchiseptica*, *B. pertussis* and *B. parapertussis*. The genomes of the classical bordetellae are closely related to each other with a mean between-species diversity of π = 0.00382 (range: π = 0.00317–0.00431) reflecting the relatively recent evolution of *B. pertussis* and *B. parapertussis* from *B. bronchiseptica*-like ancestors [5–7]. In contrast, despite forming a distinct clade, genomes of the species *B. holmesii*, *B. hinzii*, *B. avium* and *B. trematum* were not as closely related to each other (Fig. 1) with diversity values ranging from π = 0.08349 between *B. holmesii* and *B. hinzii* to π = 0.11107 between *B. trematum* and *B. avium* (Additional file 1: Table S2). The mean between-species diversity in clade B (π = 0.09884) was comparable to the mean between-species diversity of π = 0.09764 in clade C (*B. petrii* and *B. ansorpii*), but it was 25 times larger than the mean between-species diversity of π = 0.00382 between the classical bordetellae.

In most *Bordetella* species, within-species sequence diversity was very limited. It was extremely low in the human restricted *B. pertussis* (π = 0.00004) and *B. holmesii* (π = 0.00002), suggesting that population bottlenecks and/or subsequent rapid clonal expansion occurred in these species. In contrast, the diversity within *B. bronchiseptica* (π = 0.00289) was comparable to the mean between species diversity of π = 0.00382 in the classical bordetellae (Additional file 1: Table S2), and sequence diversity within *B. ansorpii* (π = 0.02119) and *B. petrii* (π = 0.07534) was at least one order of magnitude larger.

### Gene content and core genome

Next we determined presence or absence of genes in a multi-pair-wise comparison. Genes were scored as present if their predicted proteins showed ≥ 75 % identity. Based on this cut-off, we identified a total of 30,067 different genes, of which 15,636 genes were shared between at least two genomes. The remaining 14,431 genes were uniquely present in any one genome, on average 50 genes (range 1–312) in *B. bronchiseptica*, 67 (range 5–278) in *B. pertussis*, 76 and 139 in *B. parapertussis*, 54 (range 4–120) in *B. holmesii*, 142 in *B. hinzii* (range 85–242), 86 (20–141) in *B. trematum*, 1242 (range 830–1,620) in *B. petrii* and 1169 and 1183 in *B. ansorpii*. The single *B. avium* genome possessed 789 unique genes, likely a mix of species-specific and strain-specific genes.

The 850 genes that were found present in all 128 genomes (Table 2) encode essential cell structures (e.g. ribosomal protein genes) and core cell functions including energy metabolism (e.g. ATP synthase), synthesis of amino acids and nucleotides or sugar metabolism. To be considered a component of the core genome, a gene was required to be present in all 128 genomes. To account for genes that are present but might appear absent due to mis-annotation, mis-assembly, contig breaks, pseudogenes because of frameshift mutations and insertion of IS elements, we also determined the number of genes shared among 127/128 (1256 genes) and among 126/128 genomes (1462 genes), which represent the most conserved core genome in the genus.

One thousand two hundred nine genes were shared among the 34 genomes of non-classical bordetellae, and an additional 366 genes (total of 1575 genes) were shared amongst the genomes of the four non-classical *Bordetella* species *B. holmesii*, *B. hinzii*, *B. avium* and *B. trematum* (Table 2) that formed clade B in the phylogenetic tree (Fig. 1a). Consistent with the SNP-based analysis, the three species of the classical bordetellae were more closely related, sharing 1828 genes present in all 94 analyzed genomes (Table 2).

### Phylogeny based on gene content

The 15,636 genes shared between at least two genomes were used to generate a phylogenic tree that was based on gene presence or absence (Fig. 1b). The general topology of the resulting gene content-based tree was similar to that of the SNP-based tree (Fig. 1a), as it displayed the same 3 species clusters, namely clade A composed of the classical bordetellae *B. bronchiseptica*, *B. parapertussis* and *B. pertussis*; clade B containing *B. hinzii*, *B. holmesii*, *B. avium* and *B. trematum*; and clade C with *B. ansorpii* and *B. petrii*. However, there was considerably more differentiation between the genomes of the different species of the gene content-based tree than the very limited within-species differences in the SNP-based tree (with the exception of *B. petrii* and *B. ansorpii*). The gene content-based diversity among the non-classical *Bordetella* species (mean: 0.2521; range 0.1775–0.3464) was consistently 2.5 times higher than in the sequence-based tree (mean: 0.1077; range 0.0835–0.1299) (Additional file 1: Table S3, Table S4). In contrast, the gene content-based diversity between the classical *Bordetella* species (mean: 0.1078; range 0.0745–0.1311) was almost 30 times higher than the sequence-based between-species diversity (mean: 0.0038; range 0.0032–0.0043), reflecting the extremely low sequence diversity in *B. pertussis*.

### Correlation between genome wide sequence diversity and gene content

Mantel regression coefficients were calculated in order to estimate the similarity between sequence diversity and gene content. Matrices of pair-wise distances were strongly correlated (Mantel correlation coefficient = 0.86, *P* < 0.00001) between the sequence-based and gene content-based phylogenies; 74 % of the variation in the sequence-based phylogenetic tree can be explained by a linear relationship with variation in the gene content-based tree (*R*^2^ = 0.74) (Table 2, Additional file 1: Figure S2). However, this strong correlation was based mainly on differences among species, because the correlation was considerably lower within most species, ranging from *R*^2^ = 0.68 in *B. pertussis* to *R*^2^ = 0.09 in *B. holmesii*. Accordingly, sequence diversity and gene content were strongly correlated in the 34 genomes from 6 species of the non-classical bordetellae (*R*^2^ = 0.81), but the correlation was weaker in the 94 genomes from the 3 classical *Bordetella* species (*R*^2^ = 0.28). Thus, the sequence-based and the gene content-based phylogenies seem to be quantitatively comparable in *Bordetella* species, both revealing the same three clusters of *Bordetella* species (Fig. 1). With the exception of *B. petrii* and *B. ansorpii*, the within-species diversity of the *Bordetella* species was very limited, but there was considerable diversity in gene content between the individual genomes, partially owing to the presence of multiple different prophages and insertion elements.

### Large scale gene loss during speciation

**Fig. 2:**
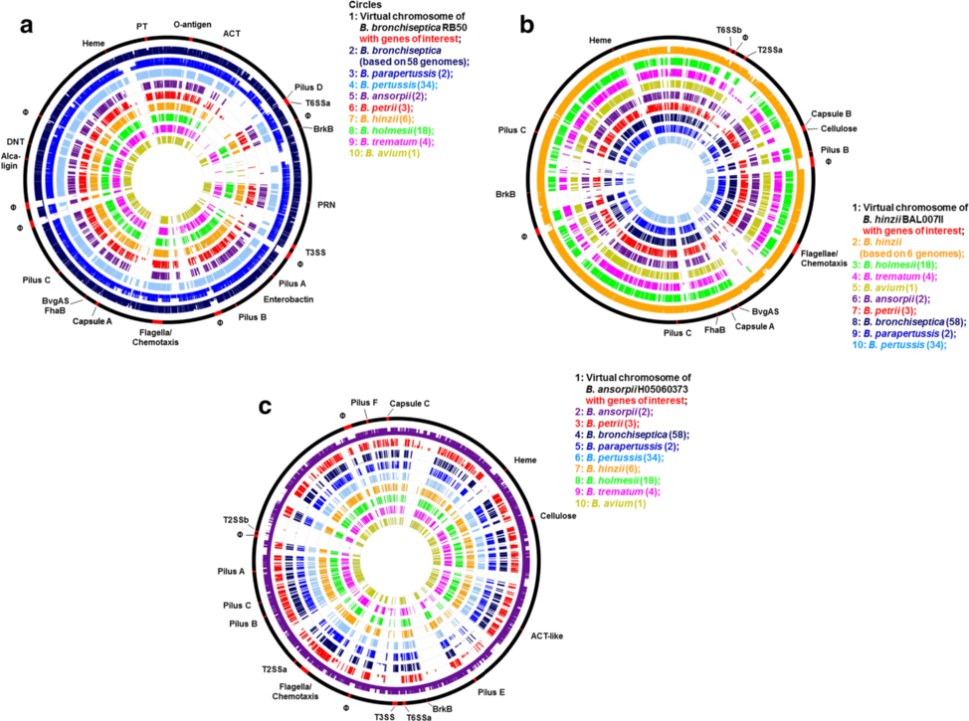
Presence and absence of genes in 128 genomes from 9 *Bordetella* species in comparison to reference genomes of **a** *B. bronchiseptica* strain RB50; **b** *B. hinzii* strain OH87 BAL007II; **c** *B. ansorpii* strain H050680373. Circle content from outside to inside: (1) Virtual chromosome of the reference genome with key factor genes or gene clusters in red. (2–10) Proportion of genes present in individual genomes per species color-coded by species. A thin line for each gene indicates the percentage of genomes in each species containing this gene. Φ - prophage

Relative to the genome of *B. bronchiseptica*, a comparison of the gene content revealed the absence of hundreds of genes distributed in clusters around the genomes of the classical bordetellae *B. pertussis* and *B. parapertussis* (Fig. 2a). Out of the 3284 core genes that are present in all 58 analyzed *B. bronchiseptica* genomes (Table 2), 694 genes (21 %) are missing from all 34 *B. pertussis* genomes. This massive gene loss is also reflected in the smaller size of *B. pertussis* genomes in comparison to *B. bronchiseptica* (Table 1). In contrast, out of the 3284 *B. bronchiseptica* core genes, only 87 genes (2.6 %) are missing from the genomes of the two *B. parapertussis* strains, 62 of which are among the genes missing from all *B. pertussis* genomes. Many of those 694 genes are involved in regulation of gene expression (~80 transcriptional regulator genes), transport (~120 transporter genes) and metabolism of a wide range of compounds (e.g. genes encoding isomerases, [de-]hydrogenases, oxidoreductases and transferases), while the function of others is unknown (~160 hypothetical proteins). Other genes lost from *B. pertussis* that are present in most *B. bronchiseptica* strains include important virulence-associated factors such as the O-antigen synthesis locus and a type VI secretion system (see below). Strikingly, many of these lost genes are also frequently missing from genomes of non-classical *Bordetella* species (Fig. 2a).

**Fig. 3:**
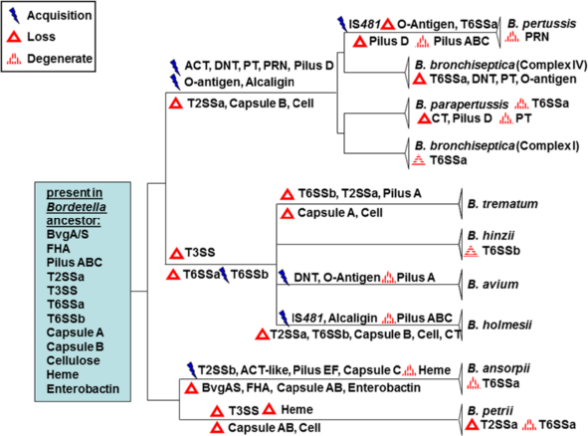
Schematic of large scale loss and limited acquisition of virulence-associated factors during the evolution and speciation of the genus *Bordetella*. The evolution of *Bordetella* into three species clades was accompanied by clade-specific, species-specific and strain-specific acquisition and loss of pathogenicity-associated factors. Gene gain or loss indicated on the branches of the tree affected the whole clade or species, events indicated below the species name affected individual strains. Cell – cellulose synthesis; CT – Chemotaxis

Similar to the classical bordetellae, substantial gene loss from the genomes of the species in clade B (*B. hinzii*, *B. trematum*, *B. avium* and *B. holmesii*) is also apparent, reflecting the large variation in their genome size. For example, the genome of *B. hinzii* is over 1 Mb larger than that of *B. avium* or *B. holmesii* (Table 1), containing 1068 *B. hinzii* core genes that are not present in either of the other two species. Analogous to *B. pertussis*, gene loss mainly affected genes encoding hypothetical proteins (~200), transcription and response regulators (~170), various transporters (~190) and enzymes involved in the metabolism of many different compounds (~380), but also outer membrane proteins such as siderophore receptors. In addition, *B. holmesii* genomes lost type II and type VI secretion systems, a capsule synthesis locus, and a large part of a flagella synthesis/chemotaxis locus (Fig. 2b, Fig. 3; see below).

**Fig. 4:**
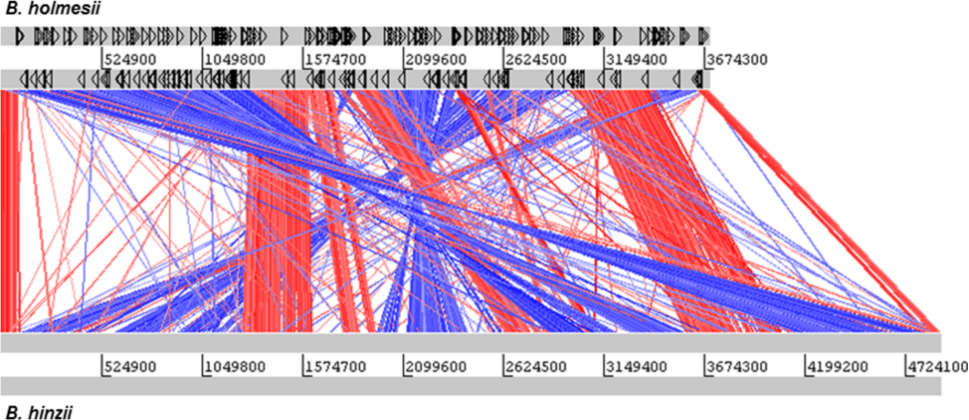
Genome comparison of *B. holmesii* and *B. hinzii*. Protein similarity (TBLASTX) between the genomes as red lines (direct matches) and blue lines (inverted matches). The gray bars represent the forward and reverse DNA strands, black triangles denote IS elements (ISEs). Many chromosomal break points are flanked by ISEs suggesting genome reduction via ISEs

In contrast to *B. hinzii*, the considerably smaller genome of *B. holmesii* contains numerous insertion sequence elements (ISEs), particularly IS*L3* (78 copies), IS*407* (64 copies) and IS*481* (51 copies). A linear genomic comparison (Fig. 4) shows 36 major genomic changes (rearrangements and deletions larger than 20 kb) in *B. holmesii* in comparison to *B. hinzii*, 28 (78 %) of which are flanked by ISEs. This suggests that acquisition, expansion and subsequent recombination between these perfect DNA repeats may have contributed to genome reduction and genomic rearrangements in this emerging species.

### Species-specific presence or absence of potential virulence-associated genes

To assess the distribution of virulence-associated genes in the *Bordetella* genus we compared the gene content in all species against that of *B. bronchiseptica* strain RB50 (Fig. 2a), *B. hinzii* strain OH87 BAL007II (Fig. 2b) and *B. ansorpii* strain H05060373 (Fig. 2c). We were particularly interested in genes potentially involved in bacteria-host interaction and pathogenicity to identify differences that could possibly explain the different virulence profiles and host ranges of these organisms. We assumed that genes with orthologues in at least two of the three *Bordetella* clades were present in the ancestor of the genus. In contrast, genes present only in a single clade or species were likely acquired in this lineage or species.

#### BvgA and BvgS

The expression of *Bordetella* virulence genes is regulated by a two-component system composed of BvgS and BvgA. The *bvgA* and *bvgS* genes are located adjacent to each other on the bacterial chromosome. The BvgA proteins share 72–75 % protein identity between the classical bordetellae and those from clade B while BvgS proteins are less conserved with only about 45 % identity. In *B. petrii*, the BvgS protein is apparently truncated as the periplasmic domain known from the other bordetellae [33] is encoded by an independent gene (locus Bpet4470), separated from the *bvgS* gene (locus Bpet4472) by *bvgA* (locus Bpet4471). An additional, truncated *bvgS* allele (locus Bpet4469) is located in reverse orientation next to this gene cluster. While *bvgA* from *B. petrii* shares 42 % to 44 % predicted protein identity with those from other *Bordetella* species, it is more closely related to genes encoding BvgA-like proteins from other *Betaproteobacteria* such as *Cupriavidus taiwanensis* (56–61 % protein identity) and a variety of *Burkholderia* species (51–57 %). Unexpectedly, no homologues of *bvgA* or *bvgS* genes were found in either of the two *B. ansorpii* genomes (Table 3).

**Table 3:** Presence and absence of specific virulence-associated key factors in the genomes of 9 *Bordetella* species

Key factor\Species	*B. bronchiseptica*	*B. parapertussis*	*B. pertussis*	*B. holmesii*	*B. hinzii*	*B. avium*	*B. trematum*	*B. petrii*	*B. ansorpii*
BvgA/BvgS/FHA	+	+	+	+	+	+	+	+	-
DNT	45/58	+	+	-	-	+	-	-	-
T1SS-ACT	55/58	+	+	-	-	-	-	-	-
T2SSa	-	-	-	-	+	+	-	2/3	+
T2SSb	-	-	-	-	-	-	-	-	+
T2SSc	-	-	-	-	-	-	-	-	1/2
Type IV Pilus A	+	+	d	d	+	d	-	+	+
Type IV Pilus B	+	+	d	d	+	+	+	+	+
Type IV Pilus C	+	+	d	d	+	+	+	+	+
Type IV Pilus D	+	1/2	-	-	-	-	-	-	-
Type IV Pilus E	-	-	-	-	-	-	-	-	+
Type IV Pilus F	-	-	-	-	-	-	-	-	+
T3SS	+	+	+	-	-	-	-	-	+
T4SS-Pertussis Toxin	42/58	d	+	-	-	-	-	-	-
T5SS-Pertactin	+	+	+	-	-	-	-	-	-
T6SSa	51/58	+	-	-	-	-	-	+	+
T6SSb	-	-	-	-	5/6	+	-	-	-
T6SSc	-	-	-	-	-	-	-	1/3	-
O-antigenA (*wbm* locus)^a^	51/58	1/2	-	-	-	-	-	-	-
O-antigenB (BAV0081-89)	-	-	-	-	-	+	-	-	-
Capsule A	+	+	+	+	+	-	-	-	-
Capsule B	-	-	-	-	+	+	+	-	-
Capsule C	-	-	-	-	-	-	-	-	1/2
Cellulose synthesis	-	-	-	-	+	+	+	-	+
Flagella	+	1/2	+	-	+	+	+	+	+
Alcaligin receptor	+	+	+	+	-	-	-	-	-
Heme receptor	+	+	+	+	+	+	+	-	d
Enterobactin receptor	+	d	+	+	+	+	+	+	-

*d* degenerate, likely not functional

^a^- *B. trematum* and *B. ansorpii* may potentially contain other, additional O-antigen synthesis loci

#### Filamentous hemagglutinin (FHA)

FHA is an important adhesin in the classical bordetellae the expression of which is regulated by BvgAS. The *Bordetella* genomes encode three different types of FHA that are so divergent that they share only 35 % protein identity. These three types are specific for each of the three *Bordetella* clades and contain protein domains that are conserved among the types such as a carbohydrate-dependent hemagglutination activity site and a hemagglutinin repeat domain as well as domains specific for each clade. Similar to *bvgAS*, *B. ansorpii* isolates do not possess *fhaB* (Table 3), and the hemagglutinin genes present encode proteins that are very different and more closely related to other *Betaproteobacteria* and even *Gammaproteobacteria* such as *Pseudomonas* than to FHA from other bordetellae, suggesting a different evolutionary origin and acquisition by horizontal gene transfer (HGT).

#### DNT

One of the toxins of the classical bordetellae whose expression is regulated by BvgAS is DNT. Although present in all three classical species, the *dnt* gene is present as a pseudogene in *B. parapertussis*_ov_ due to a frameshift mutation and it is absent from the genomes of 13/21 *B. bronchiseptica* complex IV strains. Examination of the flanking nucleotides revealed that loss of the *dnt* gene in those *B. bronchiseptica* genomes can be attributed to two independent deletion or recombination events and subsequent expansion of the respective clones. Accordingly, *dnt*-deficient strains form two clusters in the genome-based phylogenetic tree (not shown). Besides the classical bordetellae, DNT is also encoded in *B. avium* [25]. However, the predicted protein sequences of the two toxins are only 41 % identical, and the genes are located at different sites on the bacterial chromosome, suggesting that they are not true orthologues but may have been acquired independently from a currently unknown source.

#### Protein secretion systems

In the bordetellae and other Gram-negative bacteria, export of selected proteins from the cytoplasm to the exterior of the cell is carried out by six distinct secretion machineries, known as Type I-VI secretion systems. A given bacterium can possess one or more of each type of secretion system. In many bacterial pathogens, protein secretion systems themselves are regarded as important virulence factors or are involved in transport of adhesins, toxins or other effector proteins.

#### Type I secretion - Adenylate cyclase toxin (ACT)

The adenylate cyclase toxin hemolysin gene cluster consists of five genes that encode a bifunctional hemolysin/adenylate cyclase (*cyaA*), a cyclolysin activating acyltransferase (*cyaC*), a RTX-I toxin ATP-binding translocator (*cyaB*), a hemolysin secretion protein (*cyaD*), and a TolC-like outer membrane protein (*cyaE*). This gene cluster is present in genomes of only *B. bronchiseptica*, *B. pertussis* and *B. parapertussis* (Fig. 2a, Table 3), suggesting import of this gene cluster into the ancestor of the classical bordetellae before further speciation. In addition, it is absent from 3/58 *B. bronchiseptica* strains, in agreement with the previous observation that in some strains the *cya* locus was replaced by an operon predicted to encode peptide transport proteins [46]. A somewhat similar gene cluster is present in a different chromosomal location in *B. ansorpii* (Fig. 2c) containing genes predicted to encode cyclolysin, a RTX-I toxin, a hemolysin secretion protein, and a TolC-like outer membrane protein precursor. However, these predicted *B. ansorpii* proteins show less than 36 % identity to those of the classical bordetellae, but are 72–84 % identical to those from *Pseudomonas veronii* of the *Gammaproteobacteria* indicating that these two gene clusters are not orthologous and may have been acquired from different sources with diverse evolutionary origins.

#### Type II secretion system (T2SS)

Gram-negative bacteria use the type II secretion system, which is typically composed of 12 to 16 different proteins, to transport a large number of secreted proteins from the periplasm into the extracellular environment. We identified a T2SS (T2SSa) in the genomes of *B. hinzii*, *B. avium* (BAV0332-BAV0343), *B. ansorpii* and in two of the three *B. petrii* genomes (Fig. 2b, Table 3). Its presence in species from two of the three clades implies that this putative T2SS variant was included in the gene pool of the *Bordetella* ancestor and that it was subsequently lost in *B. holmesii* and *B. trematum* as well as in the lineage leading to the classical bordetellae (Fig. 3). The genomes of *B. ansorpii* harbor one (T2SSb) or two (T2SSb and T2SSc) additional T2SS elsewhere in the genome (Fig. 2c).

#### Type IV Pilus (T4P)

Type IV pili of Gram-negative bacteria are involved in adherence. They employ a modified version of the type II system for their biogenesis, and some proteins may be shared between a pilus complex and the type II system. There are several potential T4P gene clusters in the *Bordetella* genomes, one of which (Pilus A: *B. bronchiseptica* RB50 loci BB1826-BB1834) is shared among the genomes of all species with the exception of *B. trematum*, which appears to have lost it. In addition, this locus is degenerate in *B. avium* (BAV2525A-BAV2534) [25] and *B. holmesii* due to the presence of several pseudogenes. Two other T4P loci (Pilus B: BB2093-BB2100 and Pilus C: BB3192-BB3199) are shared between all *Bordetella* species but are also degenerate in *B. holmesii*, and all three loci in *B. pertussis* contain pseudogenes due to frameshifts. *B. bronchiseptica* and *B. parapertussis*_ov_ possess a further T4P locus (Pilus D: BB0776-BB0792) absent from *B. pertussis* and the genome of *B. parapertussis*_hu_ suggesting acquisition by the ancestor of the classical bordetellae and subsequent loss by those lineages. The *B. ansorpii* genomes harbor two further T4P loci (Pilus E, Pilus F) which do not have orthologues in any other bordetellae. Instead, proteins of the Pilus F locus are 80–90 % identical to those from the distantly related *Betaproteobacteria Thauera linaloolentis* of the order *Rhodocyclales*, suggesting acquisition via HGT.

#### Type III secretion system (T3SS)

Many Gram-negative bacterial pathogens use type III secretion systems, which are composed of more than 20 proteins, to deliver type III effector proteins into eukaryotic host cells (e.g. [47]). Gene clusters encoding a T3SS (*bspC*-*bspW*, *bopB*, *bopD*, *bopN*) are present in the classical bordetellae (BB1608-BB1639) as well as in *B. ansorpii*, but absent from genomes of all other species (Table 3, Fig. 3). However, while *B. parapertussis*_ov_ appears to contain an intact T3SS, two of the genes in *B. parapertussis*_hu_ are present as pseudogenes (loci BPP2215 and BPP2241), likely affecting T3SS function [5]. The T3SSs in the classical bordetellae and in *B. ansorpii* are sufficiently similar to imply a common evolutionary origin but also different enough to exclude recent HGT between the two. Thus, the T3SS was likely present in the *Bordetella* ancestor and was subsequently lost in *B. petrii* and in the ancestor of clade B.

#### Pertussis toxin (PT) and the associated type IV secretion system (T4SS)

Among the T4SSs that are present in genomes of the bordetellae, the T4SS of the pertussis toxin is the best characterized [48]. The genes encoding PT and the adjacent T4SS involved in its export are present in the genomes of only the classical bordetellae (Fig. 2a), suggesting that this gene cluster has been acquired by HGT by the ancestor of this lineage (Fig. 3). However, while all 37 genomes of *B. bronchiseptica* complex I possess this gene cluster, it is present in only 24 % (5/21) of strains from complex IV, suggesting ongoing gene loss (Fig. 2a, Fig. 3, Table 3). In addition, PT is not expressed in *B. parapertussis*_hu_, possibly because the *ptxB* gene harbors a nonsense mutation [5, 7].

#### Type V secretion system (T5SS) – autotransporters

Autotransporters are multidomain proteins that contain a translocator (or autotransporter) domain that forms a pore in the outer membrane through which the passenger or outer membrane domain of the protein is transported. With the exception of the closely related classical *Bordetella* species, that share most of the 21 autotransporter genes identified in the genome of *B. bronchiseptica* RB50 (20 in *B. parapertussis*_hu_, 16 in *B. pertussis* strain Tohama I) [5], every species contains its own set of autotransporters. The non-classical species encode considerably fewer autotransporters: 9 in *B. avium* (7 of which are intact) [25], 8 in *B. hinzii*, 6 in *B. trematum*, 4 in *B. holmesii*, 4 in *B. petrii* and 2 in *B. ansorpii*. With the exception of the classical bordetellae, orthologous autotransporters are rarely present in more than one species. Among the seven intact autotransporters identified in *B. avium* [25], only a single gene (BAV1641) has orthologues in other species. The encoded serine protease is shared with the closely related *B. hinzii* and *B. trematum*, but it is missing in *B. holmesii*. The protein has 62 % identity between those species and is a true orthologue at the same chromosomal location. In addition, *B. trematum* genomes also possess an intact adjacent autotransporter gene which is a pseudogene in *B. avium* (BAV1640) and is missing in *B. hinzii*.

Even within species, the presence of autotransporter genes is variable. For example, only 34/58 *B. bronchiseptica* genomes possess the *bapA* gene (BB1649), and 26/58 strains possess the autotransporter gene at locus BB0821. Moreover, the *brkA* gene (BB0961) is likely not functional in 9/54 isolates because of frameshifts (in 4 isolates) and a variety of amber point mutations (in 5 isolates). Another example is the highly immunogenic adhesin pertactin (BB1366) that is a pseudogene in 14 of 34 *B. pertussis* strains due to insertion of insertion sequence element IS*481*, an amber stop codon in the outer membrane portion of the gene, or a 28 amino acid deletion in the PRN signal peptide.

#### Type VI secretion system (T6SS)

The type VI secretion system encodes a syringe-like apparatus that mediates injection of effectors into both eukaryotic host cells and bacterial competitors [49–51]. We identified three different sets of T6SSs in our dataset. The first T6SS gene cluster (T6SSa) is present in genomes of *B. bronchiseptica* (BB0793-BB0818) and *B. parapertussis* from the classical bordetellae as well as in genomes of *B. petrii* and *B. ansorpii* (Fig. 2a, c). T6SSa is absent from all genomes of *B. pertussis*, suggesting loss of this gene cluster along with many other genes during the evolution of this species. Even though the locus is present in *B. parapertussis*, it is likely not functional because of missing genes and pseudogenes [5, 7]. In addition, the locus is absent from 12 % (7/58) of *B. bronchiseptica* genomes (six from complex IV and one from complex I), emphasizing ongoing loss of type VI secretion in the classical bordetellae. In *B. petrii* and *B. ansorpii* genomes the T6SSa locus is split and located at two different positions: loci Bpet0519-Bpet0524 correspond to BB0793-BB0798 and Bpet4118-Bpet4101 to BB0799-BB0820. While the genomes of *B. petrii* strains DSM12804 and J49 possess the entire T6SS, strain J51 and both *B. ansorpii* genomes harbor incomplete T6SS’s which lack six genes. Besides T6SSa, *B. petrii* DSMZ12804 possesses a second T6SS (T6SSc, Table 3) at loci Bpet2445-Bpet2461 which is not found in the other two *B. petrii* genomes. Another T6SS (T6SSb) in the genomes of *B. avium* (BAV0265-BAV0283) and in 5/6 genomes of *B. hinzii* (Fig. 2b) contains 19 genes and is located at a different chromosomal location. However, T6SSb is not present in the genomes of the related species *B. trematum* and *B. holmesii*.

Based on parsimony, we propose that T6SSa was present in the ancestor of the genus *Bordetella* and subsequently lost from clade B (Fig. 3). T6SSb is present in only the genomes of *B. avium* and *B. hinzii*, implying acquisition by a progenitor followed by loss in *B. holmesii* and *B. trematum* (Fig. 3). T6SSc in *B. petrii* may have been acquired independently in this species as protein similarities to *Pusillimonas sp.*, *Alcanigenes faecalis* and several *Pseudomonas* species suggest HGT from a different evolutionary origin.

#### Flagella and chemotaxis

Flagella are involved in motility and also play an important role during attachment of the bacteria to the eukaryotic host cell. In *Bordetella* species, flagella genes and many chemotaxis genes are located in a single large locus. While the locus is present in the genomes of all strains, 27 genes whose products are involved in flagella biosynthesis and in chemotaxis are missing from all genomes of *B. holmesii*, and 22 of those genes are also missing in *B. parapertussis*_ov_ (Table 3, Fig. 2, Fig. 3).

#### O-Antigen locus

O-antigens are the outermost domains of lipopolysaccharides and in many bacterial species contribute to evasion of the host’s immune response [52–54]. We identified the *wbm* O-antigen locus in 88 % (51/58) of *B. bronchiseptica* strains and in *B. parapertussis* (Fig. 2a, Table 3), but the O-antigen locus in *B. parapertussis*_ov_ contained pseudogenes and this gene cluster was absent from the closely related *B. pertussis*. Absence of the *wbm* O-antigen locus from genomes of all of the non-classical bordetellae suggested import of this gene cluster into the ancestor of the classical bordetellae before further speciation.

The genome of *B. avium* contains a different O-antigen synthesis gene cluster (BAV0081-BAV0089) that does not exhibit similarity to the *wbm* O-antigen locus from the classical bordetellae [25] or from any other *Bordetella* species. We identified other potential O-antigen synthesis gene clusters in *B. trematum* and *B. ansorpii* which encode enzymes involved in modification of O-antigen sugars such as epimerase/dehydratases, glycosyltransferase, amidotransferase, as well as O-antigen flippase and ABC transport genes, but further research is required to determine whether these loci are indeed involved in the synthesis of O-antigens.

#### Capsular polysaccharide

Capsules contribute to the ability of pathogens to withstand host defense mechanisms, such as complement-mediated killing, and are often considered virulence factors. Gene clusters encoding type II polysaccharide capsules were identified in genomes from all *Bordetella* species, but presence of individual capsule synthesis loci seems to be lineage- and/or species-specific. Even though *B. holmesii* and *B. hinzii* possess capsule genes that are orthologous to those from the classical bordetellae (BB2918-BB2934) (Fig. 2, “Capsule A”), this locus is degenerate because the operon structure is not preserved and several genes (e.g. *wcbD*, *wcbT*) are missing. *B. hinzii* genomes contain a second capsule synthesis locus which they share with *B. avium* (BAV2635-BAV2653) and *B. trematum* (Fig. 2, Table 3, “Capsule B”). The genome of *B. ansorpii* strain H05060373 possesses a third capsule synthesis gene cluster (Table 3, “Capsule C”).

#### Cellulose synthesis

Directly adjacent to the capsular polysaccharide locus, the *B. avium* genome contains a 12-gene operon (BAV2623-BAV2634) apparently encoding biosynthesis of a cellulose-like polymer (*wssA*-*wssJ*) [25]. Both capsule and cellulose synthesis operons are present in the same genomic configuration in *B. avium*, *B. trematum*, *B. hinzii* and *B. ansorpii* suggesting a common evolutionary origin in the *Bordetella* ancestor (Fig. 2B, Fig. 3, Table 3).

#### Iron acquisition

Several genes encode putative TonB-dependent siderophore receptors, but for only few the specific iron source is known. With the exception of *B. ansorpii*, all genomes contain the *Bordetella* ferric enterobactin receptor gene *bfeA*, but it may not be functional in either *B. parapertussis* strain due to frameshift mutations. The *Bordetella bhu* locus (*hurIR*, *bhuRSTUV*) that is required for heme iron utilization [55] was identified in the classical bordetellae and also in all species from clade B. It appears to be absent in *B. petrii* and degenerate in *B. ansorpii* as the genes *hurR* and *bhuR* (encoding a heme uptake sensor protein and the heme receptor) are missing. The alcaligin siderophore biosynthesis locus (*alcABCDER*, *fauA*) [56] was present in the classical bordetellae and in *B. holmesii* but not in other *Bordetella* species. The unusually high sequence similarity which was initially detected in a *B. pertussis* microarray [57] suggests recent horizontal acquisition of this locus by *B. holmesii*.

### Presence of specific pathogenicity-associated factors and disease

All species of the classical *Bordetella* clade A cause respiratory disease as do *B. hinzii*, *B. avium* (both in birds) and *B. holmesii* (in humans). In addition, *B. hinzii* and *B. holmesii* cause septicemia in humans. In contrast, *B. ansorpii* and *B. petrii* from clade C are not known for colonization of the respiratory tract. Instead, those species are associated with otitis media (ear infection) and wound infection in immunocompromised patients (Table 1), as are *B. trematum* isolates. We performed a Principal Component Analysis (PCA) of the distribution of virulence factors among the analyzed species to unravel potential associations between the presence of known virulence-associated genes and disease outcome (Additional file 1: Figure S3). Neither the host range of the bacteria (PC1) nor the caused disease (respiratory infection *vs.* wound infection, PC2) revealed a potential relationship with presence of the analyzed virulence factors. However, PC3 separated the human-restricted species *B. pertussis* and *B. holmesii,* both of which cause respiratory disease, from the other species. While *B. holmesii* do not possess the *B. pertussis* toxins (PT, ACT, DNT), the genomes of these species are similar in many ways. Both appear to have undergone genome reduction mediated by ISEs, both possess enterobactin, heme and alcaligin receptors for iron acquisition and a capsule (type A). In both, type IV pili loci are degenerate, and neither possesses T6SS or an O-antigen locus.

### Large scale loss and limited acquisition of virulence-associated genes during the evolution and speciation of the genus *Bordetella*

On the basis of presence and absence of genes and gene clusters in the genomes, we reconstructed the evolutionary history of the genus with a particular focus on acquisition and loss of virulence-associated genes in each species, assuming that gene orthologues present in at least two of the three *Bordetella* clades were present in the ancestor. Those include genes encoding the BvgAS regulon, FHA, serum resistance protein BrkB, enterobactin and heme iron acquisition genes, three type IV pilus encoding gene clusters, a T2SS, a T3SS and a T6SS. The *Bordetella* predecessor also likely possessed a capsule operon (“Capsule B”) plus an adjacent operon for the synthesis of a cellulose-like polymer. In contrast, virulence factors that are specific for a single clade or species are assumed to be acquired in that lineage. Theoretically, they could also have been lost in the multiple other lineages, but we assume the evolutionary scenario that requires the lowest number of steps to be more likely.

Loss of genes was more prominent than gene acquisition (Fig. 3). Gene loss frequently occurred during the genome evolution of a clade or species, but also in individual genomes of any given species, emphasizing that this process is ongoing (Table 3, Fig. 3). For example, the *Bordetella* ancestor likely possessed T2SSa which was lost in the predecessor of the classical bordetellae. Its presence in *B. avium* and *B. hinzii* but not in *B. holmesii* and *B. trematum* genomes indicates loss of the system in the latter two. Similarly, the T3SS locus was likely lost from *B. petrii* as well as from the predecessor of *B. trematum*, *B. avium*, *B. holmesii* and *B. hinzii*. Likewise, T6SSa has also been lost from clade B. In addition, T6SSa is also absent from 7/58 *B. bronchiseptica* genomes and was lost by *B. pertussis* since its divergence from its *B. bronchiseptica*-like ancestor (Table 3, Fig. 3). Despite a previous study which highlighted the importance of this T6SS for pathogenicity and persistence during infection [8], the ongoing degradation of this locus is further illustrated by missing subsets of genes in *B. ansorpii* and 1/3 *B. petrii* genomes as well as by pseudogenes and gene loss in *B. parapertussis*.

Gain of novel virulence traits also seems to have occurred in multiple examples. Amongst the most notable acquisitions are well known toxins and other virulence factors of the classical bordetellae, including DNT, ACT, PT, and the O-antigen gene cluster (Table 3, Fig. 3). DNT also appears to be imported into *B. avium*, likely by an independent acquisition event. Other HGT events likely led to the acquisition of T6SSb in *B. hinzii* and *B. avium* and of T2SSb in *B. ansorpii*. HGT as such does not appear to be rare because most *Bordetella* genomes contain one or more of several different prophages. For example, two *B. trematum* isolates with almost identical nucleotide sequences possess 362 and 260 unique genes, respectively, many of which have homology to phage sequences. However, many virulence associated factors that were acquired by a predecessor of a particular species or clade, have in many instances also been lost or functionally inactivated in an entire species or in subsets of strains, emphasizing the ongoing evolution of the genus.

## Discussion

### Three clades of *Bordetella* species

Our analysis of whole genome sequence-based and the gene content-based phylogenies of the *Bordetella* genus revealed three species clades (Fig. 1). We note that we might be missing other, currently unknown *Bordetella* species representing intermediate lineages or clades. Potential candidates might be recently proposed novel *Bordetella* species isolated from human respiratory specimens [58], a *Bordetella* species from mice that is related to *B. hinzii* [59], or environmental isolates from the plaster wall surface of mural paintings in Japan [60].

The whole genome-wide SNP-based phylogeny was largely congruent with the 16S rRNA gene (*R*^2^ = 0.35) and 23S rRNA gene (*R*^2^ = 0.39) phylogenies but the position of *B. holmesii* was different (Fig. 1, Additional file 1: Figure S1). Initial comparisons of 16S rRNA sequences suggested that *B. holmesii* is more closely related to *B. pertussis* than it is to *B. avium* or *B. trematum* [28, 61], but subsequent analyses showed that *B. holmesii* likely acquired the 16S rRNA gene, part of the 23S rRNA gene and IS*481* by HGT from *B. pertussis* as comparative genomic hybridization showed substantial genomic divergence between the two species [57]. Indeed, *B. holmesii* genomes form a clade with those from *B. hinzii*, *B. avium* and *B. trematum* while the genomes of the classical bordetellae including *B. pertussis* are more distantly related (Fig. 1). Without *B. holmesii* the correlation between the genome-wide and the ribosomal RNA gene phylogenies increased to *R*^2^ = 0.43 (16S rRNA) and to *R*^2^ = 0.54 (23S rRNA), respectively.

### Functionally strongly conserved *vs.* homologous divergent genes - caveats

We chose a stringent cut-off of 75 % protein identity for the analysis of presence and absence of genes. Genes encoding proteins that met this criterion were scored as present, while genes encoding proteins with lower similarity were considered absent. This stringent cut-off worked well with the classical bordetellae which are very similar. Likewise, this cut-off worked for protein sequences of species that belonged to the same clade of non-classical bordetellae such as *B. hinzii* and *B. avium*. Accordingly, genes that were scored as absent from *B. parapertussis* and *B. pertussis* in comparison to *B. bronchiseptica* were indeed missing and have been lost during evolution and speciation. However, several true gene orthlogues were scored as different genes because they had lower similarity in protein comparisons between genomes from different clades. This resulted in the excessively high number of over 30,000 total genes in the dataset. For example, our analysis identified three clusters of BvgA and BvgS protein sequences that coincided with the three species clades, but the proteins are in fact orthlogues (Additional file 1: Figure S4). A less stringent cut-off at 50 % identity might have facilitated identification of the BvgA homologues that showed 72–75 % protein identity between the classical bordetellae and those of clade B containing *B. hinzii*, *B. holmesii*, *B. avium* and *B. trematum*, but still would have missed BvgS (45 % identity). However, lowering the cut-off increases the risk of identifying false-positives in the form of paralogues instead of homologues such as the hemagglutinins encoded by *fhaB* and its very similar paralogue *fhaS*.

Nevertheless, our stringent cut-off allowed identification of 850 genes present in all analyzed genomes, representing the most conserved core genome of the genus. Core genes encoding conserved structural components such as ribosomal proteins and metabolic key components have experienced only limited diversification. Strong purifying selection caused by functional constraints preserved most of the protein sequences, resulting in relatively low genetic distance between pairs of proteins from different clades of *Bordetella* species (Additional file 1: Figure S4). In contrast, genes encoding proteins with few functional domains were more prone to diversification due to less stringent purifying selection.

The core genome based on genome wide SNPs contained only 184,916 bp shared among all 128 genomes, theoretically sufficient to encode approximately 170 genes. However, the shared nucleotides comprise in fact stretches of 827 genes, most of which encode proteins involved in amino acid, nucleotide and sugar metabolism as well as replication, transcription and translation (e.g. ribosomal proteins). The majority of those 827 genes are among the 850 genes that are present in all analyzed genomes of the *Bordetella* genus (Table 2).

### Old species and recently emerged species

The within-species genetic diversity varies widely, from π = 0.00002 in *B. holmesii* to π = 0.07534 in *B. petrii*. In addition to efficient mutation repair, low sequence diversity implies a relatively recent origin of the species. The limited time frame for mutations to occur is reflected in the limited genetic diversity of *B. pertussis* (π = 0.00004). The global phylogeny of a worldwide collection of *B. pertussis* strains revealed two deep branches that coalesce about 2,300 years ago (median, 2,296 years; 95 % confidence interval: 1,428 to 3,340) [39]. In addition, the origin of *B. pertussis* and its specialization to the human host was associated with a severe bottleneck that strongly reduced sequence diversity and that is characterized by a dramatic loss and inactivation of hundreds of genes due to an expansion of insertion sequence elements [5]. Subsequently, one of the branches in the phylogenetic tree, that contains over 98 % of all analyzed strains, started to expand about 500 years ago [39] which correlates with the first descriptions of whooping cough outbreaks in Persia [62] and in Europe [39].

The emergence of the human respiratory pathogen *B. holmesii* is likely due to recent expansion of another successful bacterial clone. The extremely low sequence diversity among *B. holmesii* isolates (π = 0.00002), which is only half of that of *B. pertussis*, suggests another strong bottleneck associated with this species’ recent origin. The genome of *B. holmesii* is about 1 Mb smaller than that of the related *B. hinzii*, even smaller than that of *B. pertussis* (Table 1). Just like *B. pertussis*, *B. holmesii* is also restricted to the human host [14, 63], and both bacterial species have undergone genome reduction during host specialization, mediated by acquisition, expansion and recombination between ISEs. *B. holmesii* acquired a genomic island, likely from *B. pertussis*, that contains an alcaligin siderophore biosynthesis locus and antibiotic resistance efflux pumps [57]. This may enhance colonization of the host’s airways by scavenging iron and by increased resistance to host defensins and other antimicrobial peptides, and may thus be among the key events in the evolution of *B. holmesii*.

In contrast, *B. petrii* and *B. ansorpii* not only possess the largest genomes in the genus (Table 1) but also exhibit the greatest genetic diversity (Table 2). The sequence diversity of their genomes is at least one order of magnitude larger than that of the other species, suggesting that *B. petrii* and *B. ansorpii* are older than other *Bordetella* species, which is consistent with their position in the phylogenetic tree, close to the root of the genus.

Many genes that were lost from *B. pertussis* in comparison to its *B. bronchiseptica*-like ancestor are also absent from the genomes of the non-classical *Bordetella* species (Fig. 2a). Consequently, the gene content of *B. pertussis* is more similar to that of the non-classical *Bordetella* species than is the gene content of *B. bronchiseptica*. This affects the pair-wise distance matrices and, as a result, *B. pertussis* seems to be ancestral to *B. bronchiseptica* in the gene content-based tree (Fig. 1b). However, the genome-wide SNP-based phylogenetic tree (Fig. 1a), the analysis of gene loss during evolution as well as previous studies [5–7] clearly identified *B. bronchiseptica* as the predecessor of the classical bordetellae.

### Ancient and recent loss of virulence associated genes

Several studies reported a recent increase in the prevalence of *B. pertussis* strains that are deficient in PRN expression [64–67], and our analysis also revealed the disruption of *prn* genes by insertion elements of the IS*481* family. The strongly immunogenic PRN is an important component of the currently used acellular pertussis vaccine, and functional inactivation of PRN expression seems to support the opinion that the evolution of *B. pertussis* may be driven by evasion of, or adaptation to, vaccine-mediated immunity [39, 68]. However, PRN is not the only virulence associated factor that is lost and/or inactivated in individual strains of a species. For example, two deletion events led to loss of the *dnt* gene in 13/58 *B. bronchiseptica* strains. In addition, T6SSa is missing in 7 and degenerate in 3 *B. bronchiseptica* strains as well as in *B. parapertussis* (Fig. 3). All these observations emphasize the ongoing loss of virulence-associated factors in the classical bordetellae.

Gain and loss of virulence-associated factors is not limited to the classical *Bordetella* species, but appears to be a pattern throughout the evolution of the genus. Besides individual strains, entire species as well as whole clades have repeatedly experienced gene acquisition and loss, including loci encoding type II, type III and type VI secretion systems, capsules, cellulose synthesis, O-antigen, pili, and toxins such as ACT, DNT and PT (Fig. 3). Interpreting the evolution of individual species in the context of the evolution of others of the same genus will contribute important perspective to the various theories about the driving forces for evolution, new and old. In this regard, the evolution of *B. pertussis* should be considered in the context of the evolution of other bordetellae and the various evolutionary pressures that drive gene gain and loss.

## Conclusions

In this study we analyzed the genetic diversity and gene content in multiple genomes from nine *Bordetella* species. The sequence-based and gene content-based phylogenies are largely congruent among the species, indicating co-evolution of sequence diversity and gene content in the genus. We analyzed the evolution of the entire genus with particular focus on the acquisition and loss of virulence-associated genes and show that gain and loss of multiple genes, including those encoding bacterial toxins, protein secretion systems and other virulence-associated factors, shape the diversification and speciation in the genus. Gene loss was more frequent than gene acquisition, and recent loss and functional inactivation of genes, including those encoding pertussis vaccine components and bacterial toxins in individual strains, emphasize ongoing evolution. However, gene loss is not a new phenomenon in *Bordetella* but appears to be a pattern throughout the evolution of the genus suggesting that current genomic changes in *B. pertussis* isolates should be considered in the context of the evolution of the entire genus and of the various factors that drive gene gain and loss, of which vaccines are only one.

## Methods

### Genome sequences

A detailed list of the analyzed 128 genomes from all 9 *Bordetella* species is provided in Additional file 1: Table S1. The table the host from which the bacterium was isolated, the NCBI genome accession number and the reference for the original genome publication. To sequence *B. ansorpii* and *B. trematum* genomes, chromosomal DNA was sheared and size-selected to 150–250 bp, and used to produce Illumina indexed sequencing libraries according to manufacturer’s instructions. The samples were pooled and sequenced (with other unrelated samples) on a single Illumina HiSeq 2000 lane to produce 100 bp paired-end reads. Each sample was sequenced to a depth of between 200 and 333-fold. The reads were assembled using Velvet to produce between 19 and 42 contigs with an N50 of between 371,935 and 615,230 bp, and a maximum contig size of between 646,753 and 2,049,015 bp.

### Genome-wide SNP-based tree

Each genome sequence was processed into a 54-bp paired-end DNA library simulating Solexa sequencing reads. Using an alignment-free method of comparing DNA sequences, implemented in SSAHA2 version 2.5.4 [69], each processed genome was mapped onto the reference genome of *B. bronchiseptica* strain RB50 [5] with the following parameters: k-mer 13, a minimum Smith-Waterman score of 30 of the exact match, and step size 2. The resulting multiple sequence alignment of 5,339,179 bp in length was stripped from nucleotide positions containing sequence gaps in any of the aligned genomes, resulting in 184,916 bp shared among all 128 genomes. Subsequently, a distance matrix was calculated in R [70], and used to visualize the tree (Fig. 1a) in MEGA [71].

### Gene content-based tree

Clusters of orthologous proteins were generated in PanOCT as previously described [72, 73] using default parameters. PanOCT considers conserved gene neighborhood (CGN) in a weighted scoring scheme (thus taking into account presence/absence of neighboring genes) and the BLAST score ratio to effectively generate non-paralogous gene clusters. The program was run using default settings to cluster genes into ortholog sets. The resulting presence/absence of genes was used to generate a distance matrix in R [70], and the tree (Fig. 1b) was visualized in MEGA [71]. Presence or absence of the analyzed genes was confirmed by pair-wise genome comparisons using blastn (for closely related species such as the classical bordetellae) or tblastx (for less closely related species such as *B. hinzii* and *B. holmesii*) and visualization using the Artemis Comparison Tool (ACT) [74].

The Mantel correlation between the similarity matrices was calculated in R [70] and MS Excel. Presence and absence of genes in genomes from all 9 species in comparison to reference genomes from *B. bronchiseptica* RB50 (Fig. 2a), *B. hinzii* BAL007II (Fig. 2b) and *B. ansorpii* H05060373 (Fig. 2c) was plotted using GenomeViz [75]. Principal Components of the distribution of virulence-associated factors were calculated in R.

### Data access

The new genome sequences are available from GenBank under the accession numbers LT546645-LT546646, FKBS01000000, FKBR01000000 (*B. trematum* strains H044680328, H064000408 and NCTC12995, respectively), FKIF01000000 and FKBT01000000 (*B. ansorpii* strains H050680373 and NCTC13364, respectively).

## Additional file

Additional file 1: Supplementary Figures S1-S4, Supplementary Tables S1-S5, and Supplementary References. (PDF 1011 kb)
