# Supplementary material for: Acquisition and loss of virulence-associated factors during genome evolution and speciation in three clades of Bordetella species
Source: BMC Genomics. 2016 Sep 30;17:767. doi: 10.1186/s12864-016-3112-5 (PMC5045587; doi:10.1186/s12864-016-3112-5)
Supplement: Additional file 1: — Supplementary Figures S1-S4, Supplementary Tables S1-S5, and Supplementary References. (PDF 1011 kb) [file 12864_2016_3112_MOESM1_ESM.pdf]

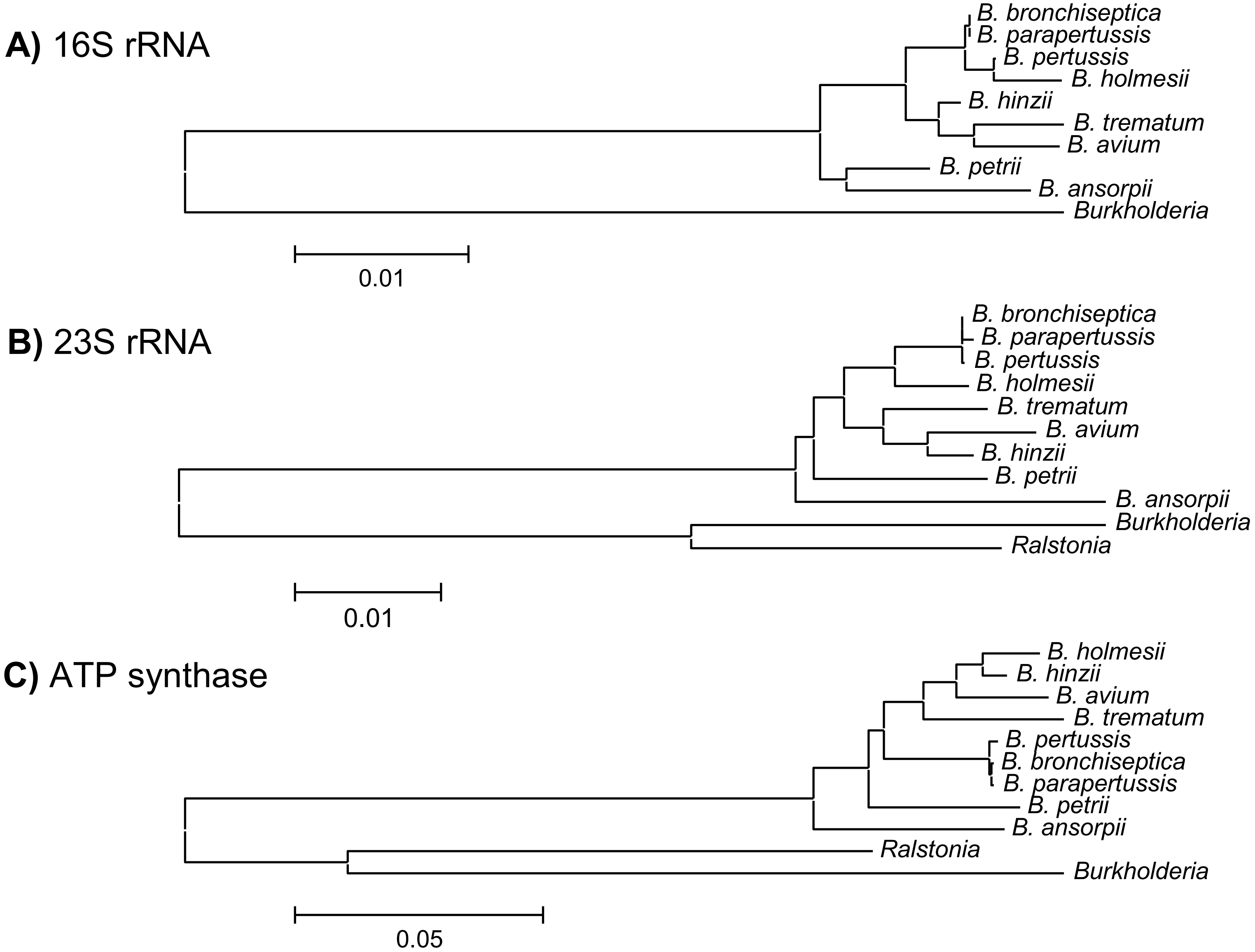

Figure S1. Phylogenetic structure (Neighbor-joining trees) of A) 16S rRNA gene sequences, B) 23S rRNA gene sequences, and C) 8 concatenated ATP synthase proteins from *Bordetella* and members of the closely related genera *Burkholderia* and *Ralstonia* as an outgroup. In all 3 trees, *B. ansorpii* and *B. petrii* are located closest to the root of the *Bordetella* species.

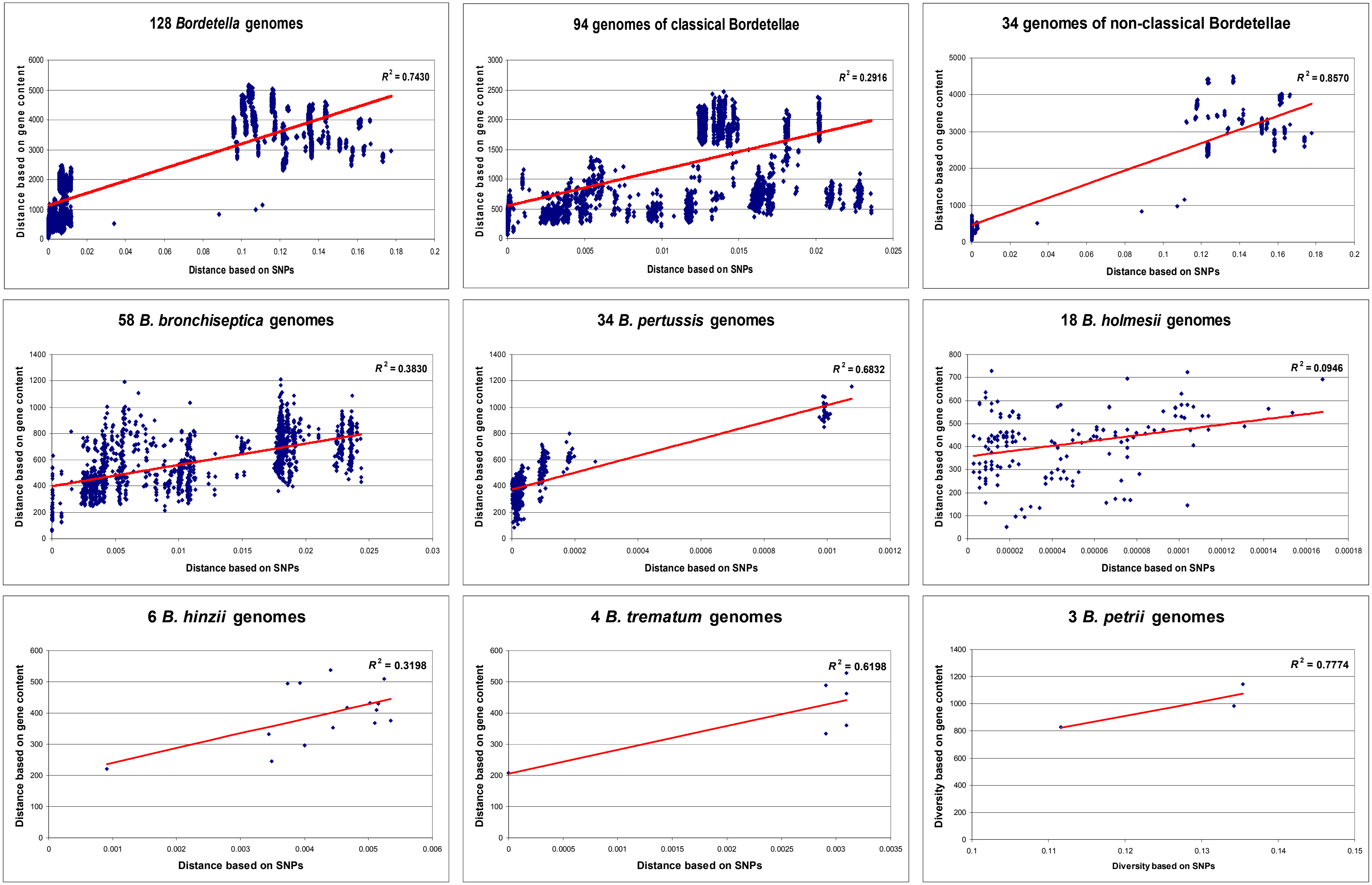

Figure S2. Correlation between sequence-based and gene content-based matrices of genetic distance between *Bordetella* genomes.

A) ATP synthase

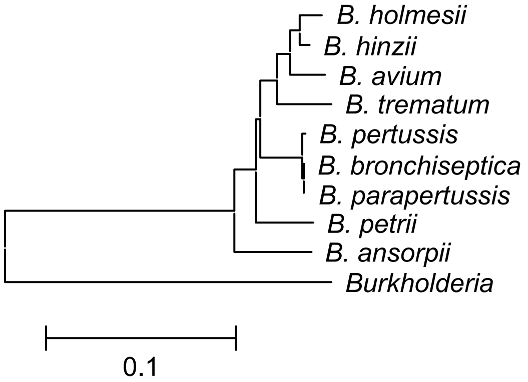

B) BvgA

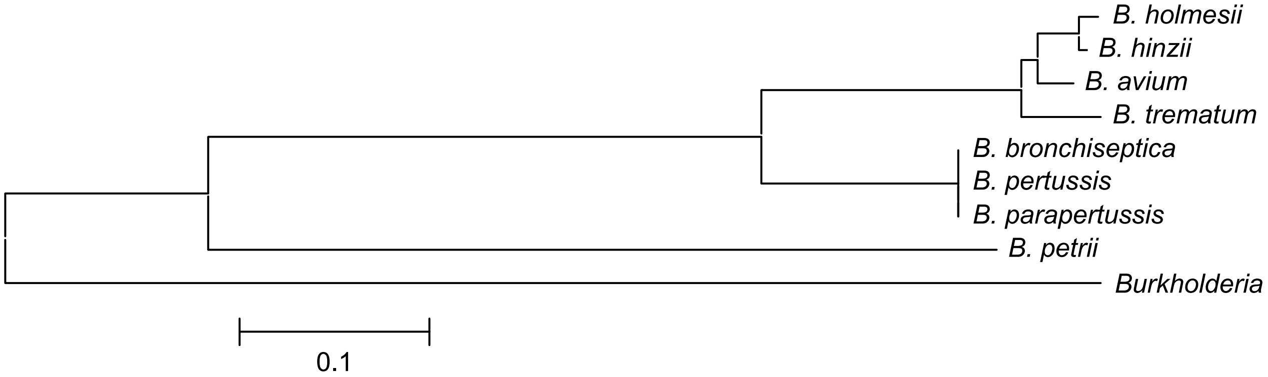

Figure S3. Phylogenetic structure (Neighbor-joining trees) of A) 8 concatenated ATP synthase proteins and B) BvgA from *Bordetella* species and from *Burkholderia pseudomallei* as an outgroup. The low average ATP synthase diversity between species clades (π = 0.0665, range = 0.553-0.0783) reflects strong purifying selection due to functional constraints. Accordingly, those genes are part of the *Bordetella* core genome. In contrast, the over 10-fold larger average BvgA diversity between species clades (π = 0.7756, range = 0.2781-1.0262) indicates that large parts of the proteins are less functionally conserved which splits BvgA protein sequences into 3 species clade-specific groups of proteins. The trees are drawn to scale.

A Species

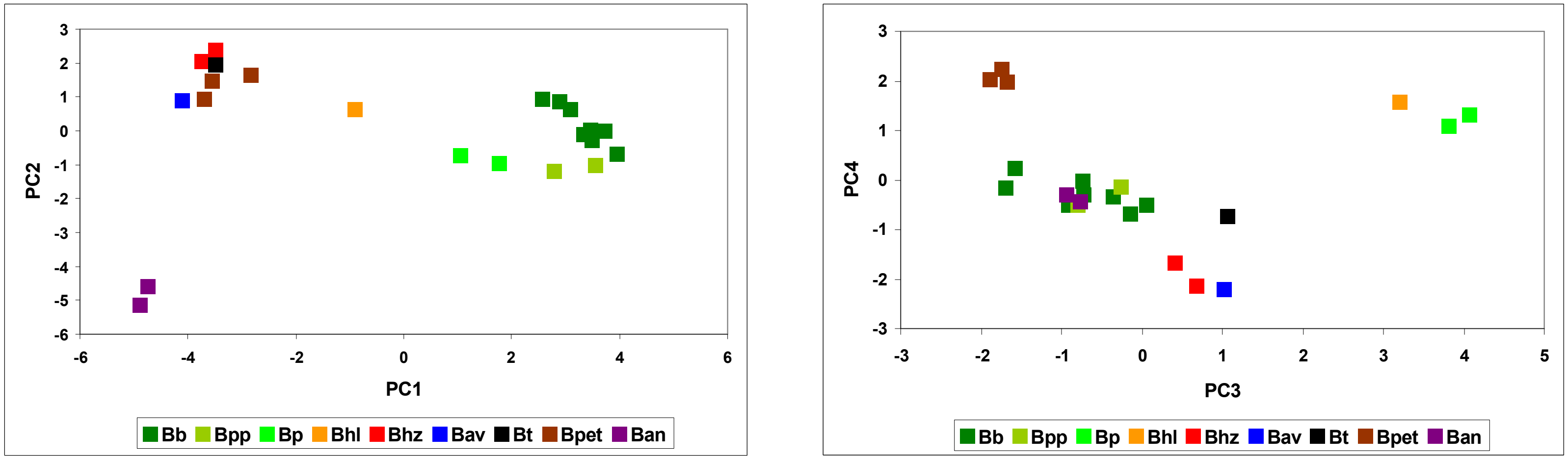

B Host and disease

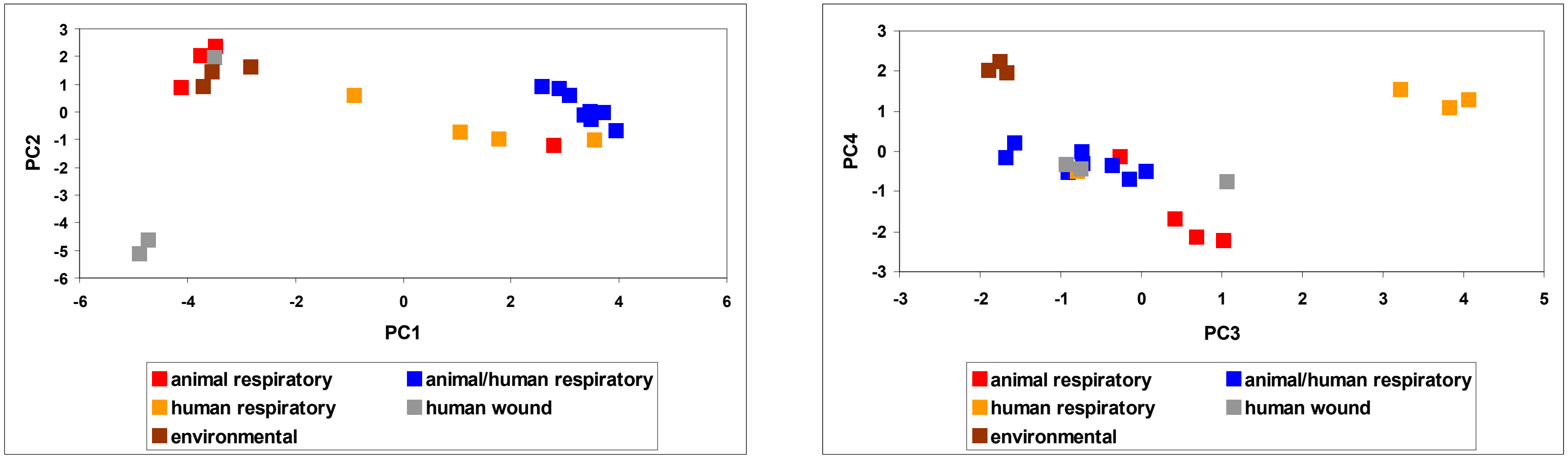

Supplementary Figure 4. Principal Component Analysis of presence/absence of virulence-associated factors in Bordetella genomes by A) *Bordetella* species; B) host and disease. The genomes from each species were grouped by presence/absence of individual factors, and any unique combination of factors was analyzed as separate data entry resulting in several data points per species. PC1 divides the classical from the non-classical species, PC2 isolates *B. ansorpii*, and PC3 separates the genomes of the human-restricted *B. pertussis* and *B. holmesii* from those of the other species. Bb *B. bronchiseptica*; Bpp *B. parapertussis*; Bp *B. pertussis*; Bhl *B. holmesii*; Bhz *B. hinzii*; Bav *B. avium*; Bt *B. trematum*; Bpet *B. petrii*; Ban *B. ansorpii*

Table S1. Genomes of *Bordetella* isolates.

**Species**	**Strain**	**Host**	**NCBI accession number**	**Comp lex**	**Reference**
B. bronchiseptica	RB50	Rabbit	BX470250	C I	[1]
B. bronchiseptica	RB630	Rabbit	JGYA00000000	C I	[2]
B. bronchiseptica	3E44	Rabbit	JGWK00000000	C I	[2]
B. bronchiseptica	A1-7	Rabbit	JGWO00000000	C I	[2]
B. bronchiseptica	B18-5	Rabbit	JGWP00000000	C I	[2]
B. bronchiseptica	B20-10725633	Rabbit	JGWQ00000000	C I	[2]
B. bronchiseptica	980	Unknown	JGWM00000000	C I	[2]
B. bronchiseptica	CARE970018BB	Pig	JGWS00000000	C I	[2]
B. bronchiseptica	MBORD849	Pig	JGXW00000000	C I	[2]
B. bronchiseptica	KM22	Pig	JNHR00000000	C I	[3]
B. bronchiseptica	MBORD595	Dog	JGXH00000000	C I	[2]
B. bronchiseptica	MBORD785	Dog	JGXU00000000	C I	[2]
B. bronchiseptica	MBORD839	Dog	JGXV00000000	C I	[2]
B. bronchiseptica	253	Dog	HE965806	C I	[4]
B. bronchiseptica	MBORD635	Cat	JGXK00000000	C I	[2]
B. bronchiseptica	MBORD782	Cat	JGXT00000000	C I	[2]
B. bronchiseptica	MBORD665	Guinea pig	JGXL00000000	C I	[2]
B. bronchiseptica	MBORD668	Guinea pig	JGXM00000000	C I	[2]
B. bronchiseptica	MBORD670	Guinea pig	JGXN00000000	C I	[2]
B. bronchiseptica	MBORD678	Guinea pig	JHBQ00000000	C I	[2]
B. bronchiseptica	MBORD762	Guinea pig	JHBR00000000	C I	[2]
B. bronchiseptica	MBORD624	Horse	JGXI00000000	C I	[2]
B. bronchiseptica	MBORD632	Horse	JGXJ00000000	C I	[2]
B. bronchiseptica	MBORD731	Horse	JGXS00000000	C I	[2]
B. bronchiseptica	M435/02/3	Seal	JGXE00000000	C I	[5]
B. bronchiseptica	M85/00/2	Seal	JGXF00000000	C I	[5]
B. bronchiseptica	SO10328	Sea otter	JGYB00000000	C I	[2]
B. bronchiseptica	1289	Monkey	CAKS01000000	C I	[2]
B. bronchiseptica	MBORD681	Koala	JGXP00000000	C I	[2]
B. bronchiseptica	MBORD698	Koala	JGXQ00000000	C I	[2]
B. bronchiseptica	00-P-2796	Human	JGWH00000000	C I	[2]
B. bronchiseptica	D756	Human	JGWT00000000	C I	[2]
B. bronchiseptica	D989	Human	JGWU00000000	C I	[2]
B. bronchiseptica	D993	Human	JGWV00000000	C I	[2]
B. bronchiseptica	E010	Human	JGWW00000000	C I	[2]
B. bronchiseptica	E012	Human	JGWX00000000	C I	[2]
B. bronchiseptica	E013	Human	JGWY00000000	C I	[2]
B. bronchiseptica	MBORD591	Dog	JGXG00000000	C IV	[2]
B. bronchiseptica	7E71	Horse	JGWL00000000	C IV	[2]
B. bronchiseptica	CA90 BB02	Turkey	JHBU00000000	C IV	[2]
B. bronchiseptica	CA90 BB1334	Turkey	JGWR00000000	C IV	[2]
B. bronchiseptica	F-1	Turkey	JGXA00000000	C IV	[2]
B. bronchiseptica	F2	Turkey	JGXB00000000	C IV	[2]

B. bronchiseptica	MBORD707	Turkey	JGXR00000000	C IV	[2]
B. bronchiseptica	MBORD901	Turkey	JGXX00000000	C IV	[2]
B. bronchiseptica	OSU054	Turkey	JHBZ00000000	C IV	[2]
B. bronchiseptica	OSU095	Turkey	JGXY00000000	C IV	[2]
B. bronchiseptica	OSU553	Turkey	JGXZ00000000	C IV	[2]
B. bronchiseptica	00-P-2730	Human	JGWG00000000	C IV	[2]
B. bronchiseptica	E014	Human	JGWZ00000000	C IV	[2]
B. bronchiseptica	F4563	Human	JGXC00000000	C IV	[2]
B. bronchiseptica	GA96-01	Human	JGXD00000000	C IV	[2]
B. bronchiseptica	MBORD675	Human	JGXO00000000	C IV	[2]
B. bronchiseptica	MO211	Human	JHOJ00000000	C IV	[2]
B. bronchiseptica	MO275	Human	JHBS00000000	C IV	[2]
B. bronchiseptica	SBL-F6116	Human	JHBT00000000	C IV	[2]
B. bronchiseptica	MO149	Human	HE965806	C IV	[4]
B. bronchiseptica	Bbr77	Human	CAKU01000000	C IV	[4]
B. pertussis	Tohama I	Human	BX470248		[1]
B. pertussis	CS	Human	CP002695		[6]
B. pertussis	STO1-CHLA-0011	Human	AXSP00000000		[7]
B. pertussis	H897	Human	AXSO00000000		[7]
B. pertussis	H918	Human	AXSN00000000		[7]
B. pertussis	H921	Human	AXSM00000000		[7]
B. pertussis	H939	Human	AXSL00000000		[7]
B. pertussis	H973	Human	AXSK00000000		[7]
B. pertussis	STO1-SEAT-0004	Human	AXSJ00000000		[7]
B. pertussis	I002	Human	AXSI00000000		[7]
B. pertussis	I036	Human	AXSH00000000		[7]
B. pertussis	I176	Human	AXSG00000000		[7]
B. pertussis	STO1-CHOC-0008	Human	AXRV00000000		[7]
B. pertussis	STO1-CHOM-0012	Human	AXRU00000000		[7]
B. pertussis	STO1-CNMC-0004	Human	AXSV00000000		[7]
B. pertussis	STO1-CHOC-0016	Human	AXSA00000000		[7]
B. pertussis	STO1-CHOC-0017	Human	AXRZ00000000		[7]
B. pertussis	STO1-CHOC-0018	Human	AXRY00000000		[7]
B. pertussis	STO1-CHOC-0019	Human	AXRX00000000		[7]
B. pertussis	STO1-CHOC-0021	Human	AXRW00000000		[7]
B. pertussis	CHLA-15	Human	AXSD00000000		[7]
B. pertussis	CHLA-13	Human	AXSE00000000		[7]
B. pertussis	CHLA-20	Human	AXSC00000000		[7]
B. pertussis	CHLA-26	Human	AXSB00000000		[7]
B. pertussis	STO1-CHLA-0006	Human	AXSF00000000		[7]
B. pertussis	CHLA-11	Human	AYXH01000000		this study
B. pertussis	B200	Human	JGWE01000000		this study
B. pertussis	2250905	Human	AXSU00000000		[7]
B. pertussis	2356847	Human	AXST00000000		[7]
B. pertussis	2371640	Human	AXSS00000000		[7]
B. pertussis	STO1-SEAT-0006	Human	AXSR00000000		[7]
B. pertussis	STO1-SEAT-0007	Human	AXSQ00000000		[7]

B. pertussis	H934	Human	JGWF00000000		this study
B. pertussis	18323	Human	HE965805		[4]
B. parapertussis	12822	Human	BX470249		[1]
B. parapertussis	Bpp5	Sheep	HE965803		[4]
B. holmesii	H620	Human	JJNW01000000		this study
B. holmesii	H572	Human	JFZY00000000		[8]
B. holmesii	H585	Human	JFZZ00000000		[8]
B. holmesii	H629	Human	JGVZ00000000		[8]
B. holmesii	H635	Human	JGAA00000000		[8]
B. holmesii	H643	Human	JGWD00000000		[8]
B. holmesii	H719	Human	JGWA00000000		[8]
B. holmesii	H785	Human	JGWB00000000		[8]
B. holmesii	H809	Human	JMGZ00000000		[8]
B. holmesii	04P3421	Human	JGWC00000000		[8]
B. holmesii	1058	Human	JDTF01000000		Tettelin[*1]
B. holmesii	30539	Human	JDFP01000000		Tettelin[*1]
B. holmesii	35009	Human	JDSK01000000		Tettelin[*1]
B. holmesii	41130	Human	JDSC01000000		Tettelin[*1]
B. holmesii	44057	Human	CP007495		Tettelin[*1]
B. holmesii	70147	Human	JDSJ01000000		Tettelin[*1]
B. holmesii	F627	Human	AOEW01000000		[9]
B. holmesii	H558	Human	AOFR01000000		[9]
B. hinzii	OH87 BAL007II	Turkey	JHEM00000000		[10]
B. hinzii	CA90 BAL1384	Turkey	JHEO00000000		[10]
B. hinzii	4161	Turkey	JHER00000000		[10]
B. hinzii	1277	Human	JHES00000000		[10]
B. hinzii	L60	Human	JHEN00000000		[10]
B. hinzii	5132	Rabbit	JHEQ00000000		[10]
B. trematum	CCUG_13902	Human	AWNL01000000		[11]
B. trematum	H044680328	Human	LT546645		this study
B. trematum	H064000408	Human	FKBS01000000		this study
B. trematum	NCTC12995	Human	FKBR01000000		this study
B. avium	197N	Turkey	AM167904		[12]
B. petrii	DSM_12804	Environmental	AM902716		[13]
B. petrii	J49	Environmental	JAEJ01000000		Gladden[*2]
B. petrii	J51	Environmental	JAEP01000000		Gladden[*2]
B. ansorpii	NCTC13364	Human	FKBT01000000		this study
B. ansorpii	H050680373	Human	FKIF01000000		this study

[*1] - Tettelin H, Hooven TA, Hine E, Su Q, Huard RC, Della-Latta P, Daugherty SC, Agrawal S, Sengamalay N, Tallon LJ, Sadzewicz L, Whittier S, Fraser CM and Ratner AJ. Whole genome sequencing of *Bordetella holmesii*, an emerging opportunistic infection of humans. unpublished.

[*2] - Gladden J, Huntemann M, Han J, Chen A, Kyrpides N, Mavromatis K, Markowitz V, Palaniappan K, Ivanova N, Schaumberg A, Pati A, Liolios K, Nordberg HP, Cantor MN, Hua SX and WoykeT. Genome sequencing of lignin-degrading bacterial isolates. unpublished.

Supplementary Table 2. Within and between species genetic distance based on genome-wide sequence diversity.

Bordetella species	Number of Genomes	Within-species genetic distance	Pair-wise between species genetic distance							
			B. bronchi-septica	*B. para-pertussis*	*B. pertussis*	*B. trematum*	*B. hinzii*	*B. avium*	*B. holmesii*	*B. ansorpii*
B. bronchiseptica	58	0.00289								
B. parapertussis	2	0.00253	0.00317							
B. pertussis	34	0.00004	0.00397	0.00431						
B. trematum	4	0.00122	0.08491	0.08576	0.08595					
B. hinzii	6	0.00121	0.07438	0.07521	0.07552	0.08488				
B. avium	1	NA	0.10385	0.10443	0.10487	0.11107	0.09226			
B. holmesii	18	0.00002	0.10186	0.10250	0.10298	0.11089	0.08349	0.11043		
B. ansorpii	2	0.02119	0.07958	0.08025	0.08087	0.10680	0.09558	0.12722	0.12557	
B. petrii	3	0.07534	0.08195	0.08267	0.08317	0.11125	0.09992	0.12995	0.12823	0.09764

Supplementary Table 3. Within and between species genetic distance based on presence and absence of genes.

Bordetella species	Number of Genomes	Within-species genetic distance	Pair-wise between species genetic distance							
			B. bronchi-septica	*B. para-pertussis*	*B. pertussis*	*B. trematum*	*B. hinzii*	*B. avium*	*B. holmesii*	*B. ansorpii*
B. bronchiseptica	58	0.03721								
B. parapertussis	2	0.07783	0.0745							
B. pertussis	34	0.02723	0.1311	0.1179						
B. trematum	4	0.02465	0.3714	0.3160	0.2849					
B. hinzii	6	0.02448	0.3575	0.3093	0.2800	0.2805				
B. avium	1	NA	0.3305	0.2752	0.2441	0.2245	0.2197			
B. holmesii	18	0.02536	0.3067	0.2551	0.2231	0.2315	0.1775	0.1849		
B. ansorpii	2	0.03161	0.4008	0.3435	0.3142	0.3464	0.3446	0.3039	0.2982	
B. petrii	3	0.06299	0.2728	0.2322	0.2076	0.2514	0.2457	0.2219	0.2066	0.2436

Supplementary Table 4. Ratio of gene content based vs sequence based within and between species genetic distances.

Bordetella species	Number of Genomes	Ratio of within-species genetic distances	Ratio of Pair-wise between species genetic distance							
			B. bronchi-septica	*B. para-pertussis*	*B. pertussis*	*B. trematum*	*B. hinzii*	*B. avium*	*B. holmesii*	*B. ansorpii*
B. bronchiseptica	58	12.9								
B. parapertussis	2	30.8	23.5							
B. pertussis	34	680.8	33.0	27.4						
B. trematum	4	20.2	4.4	3.7	3.3					
B. hinzii	6	20.2	4.8	4.1	3.7	3.3				
B. avium	1	NA	3.2	2.6	2.3	2.0	2.4			
B. holmesii	18	1268.0	3.0	2.5	2.2	2.1	2.1	1.7		
B. ansorpii	2	1.5	5.0	4.3	3.9	3.2	3.6	2.4	2.4	
B. petrii	3	0.8	3.3	2.8	2.5	2.3	2.5	1.7	1.6	2.5

Table S5. Most conserved core genome

cluster_	protein name
1	methyltransferase GidB
2	CobQ/CobB/MinD/ParA nucleotide binding domain protein
4	chromosome Partitioning Protein
7	secretion protein SecE PA4276
8	transcription termination/antitermination factor NusG
9	ribosomal protein L11
10	ribosomal protein L1
11	50s ribosomal protein L10.
12	ribosomal protein L7/L12
13	DNA-directed RNA polymerase, beta subunit
14	dna-directed rna polymerase beta' chain
20	Two-component response regulator
24	ribosomal protein S7
28	ribosomal protein L3
29	ribosomal protein L4/L1 family
30	ribosomal protein L23
31	ribosomal protein L2
32	ribosomal protein S19
34	ribosomal protein S3
35	ribosomal protein L16
36	ribosomal protein L29
37	30S ribosomal protein S17 NMB0151
40	ribosomal protein L14
41	ribosomal protein L24
42	50S ribosomal protein L5 NMB0154
43	ribosomal protein S14p/S29e
44	ribosomal protein S8
45	50S ribosomal protein L6 NMB0157
46	ribosomal protein L18
47	ribosomal protein S5
48	ribosomal protein L30
49	ribosomal protein L15
50	preprotein translocase SecY chain
53	ribosomal protein S11
54	ribosomal protein S4
55	DNA-directed RNA polymerase, alpha subunit
57	histidine triad protein
59	thiol:disulfide interchange protein dsbd precursor
62	ribosome biogenesis GTP-binding protein YsxC
63	putative cytochrome C4
64	ResB-like protein
65	cytochrome c biogenesis protein
66	diaminopimelate decarboxylase
67	iron donor protein CyaY
69	shikimate kinase I
70	3-dehydroquinate synthase
71	phosphohydrolase-associated domain protein
93	malate synthase G
99	GAF domain protein
112	Protein of unknown function (DUF502) family

114 Endonuclease/Exonuclease/phosphatase family superfamily
155 type III pantothenate kinase
156 biotin-(acetyl-CoA-carboxylase) ligase
162 penicillin-binding protein 6 precursor
163 d-alanine aminotransferase
164 conserved hypothetical protein
165 lipoyl(octanoyl) transferase
166 lipoic acid synthetase
171 copper binding periplasmic protein CusF
172 ATP-dependent protease HslVU, ATPase subunit
174 DnaK suppressor protein NMB0056
175 CobW/P47K family protein
184 PF04340 family protein
185 diaminopimelate epimerase
186 lipid A biosynthesis (KDO)2-(lauroyl)-lipid IVA acyltransferase
190 adenosylhomocysteinase
191 Membrane protein of unknown function superfamily
192 methylenetetrahydrofolate reductase (NAD(P)H)
197 5-formyltetrahydrofolate cyclo-ligase
198 putative transglycosylase
202 uracil-DNA glycosylase
230 probable aminotransferase
233 PH domain protein
234 hydroxymethylglutaryl-CoA lyase
241 PUTATIVE HYDROLASE PHOSPHATASE PROTEIN
254 16S rRNA (cytosine(967)-C(5))-methyltransferase
256 GHKL domain protein
285 preprotein translocase, SecB subunit
286 Glutaredoxin
287 Rhodanese-like domain protein
288 phosphoglycerate mutase 1 family
289 peptidase, M23 family
290 carboxy-terminal processing protease precursor
291 thiF protein NMB2062
294 thiamine biosynthesis protein thic.
295 putative lipoprotein
301 glutamate 5-kinase
305 octaprenyl-diphosphate synthase
374 peptide chain release factor 1
375 protein-(glutamine-N5) methyltransferase, release factor-specific
376 glutaredoxin-related protein
377 3-octaprenyl-4-hydroxybenzoate carboxy-lyase
487 N-acetylmuramoyl-L-alanine amidase
490 signal recognition particle protein
858 asparagine synthase (glutamine-hydrolyzing)
859 glycosyl transferase, group 1 family protein domain protein
860 glycosyltransferase, group 1 family protein
865 FAD linked oxidase, C-terminal domain protein
870 fumarylacetoacetase
876 tetratricopeptide repeat protein
877 outer membrane lipoprotein LolB
880 ribose-phosphate diphosphokinase

881 ribosomal protein L25, Ctc-form
882 aminoacyl-tRNA hydrolase
883 CAAX protease self-immunity
884 GTP-binding protein YchF
916 asmA protein VC1039 , putative
917 molybdenum cofactor sulfurase protein, putative
918 benzoate transporter
920 pantetheine-phosphate adenylyltransferase
921 methyltransferase, putative
930 3,4-dihydroxy-2-butanone-4-phosphate synthase
931 6,7-dimethyl-8-ribityllumazine synthase
932 transcription antitermination factor NusB
934 phosphatidylglycerophosphatase A
935 protein ygad.
936 orotidine 5'-phosphate decarboxylase
937 pyridine nucleotide-disulfide oxidoreductase
938 prokaryotic diacylglycerol kinase
941 60 kda chaperonin
942 chaperonin, 10 kDa
1312 molybdenum cofactor biosynthesis protein A
1313 molybdopterin biosynthesis protein MoeA
1315 molybdopterin converting factor, subunit 2
1316 molybdopterin converting factor
1317 molybdenum cofactor biosynthesis protein C
1321 AhpC/TSA family protein
1341 magnesium and cobalt efflux protein corc.
1343 PhoH family protein
1349 preprotein translocase, YajC subunit, putative
1351 S-adenosylmethionine:tRNA ribosyltransferase-isomerase
1353 uracil phosphoribosyltransferase
1356 hypothetical protein
1358 hypothetical protein
1360 3-octaprenyl-4-hydroxybenzoate carboxy-lyase
1640 HYPOTHETICAL TRANSMEMBRANE PROTEIN
1643 4-hydroxy-tetrahydrodipicolinate synthase
1644 putative lipoprotein
1692 flavin reductase
1694 imelysin
1695 cytochrome C
1697 PF07433 family protein
1698 small conductance mechanosensitive ion channel
1700 putative exported protein
1702 RNA methyltransferase, TrmH family, group 1
1703 inositol monophosphatase family protein
1704 integral membrane protein / hemolysin
1705 undecaprenol kinase, putative
1707 peptidyl-prolyl cis-trans isomerase B
1708 tetratricopeptide repeat protein
1710 DNA-3-methyladenine glycosylase
1711 acetyl-CoA carboxylase, carboxyl transferase, alpha subunit
1713 aspartate kinase, monofunctional class
1788 UvrD/REP helicase N-terminal domain protein

1791 ABC transporter, substrate-binding protein, family 5
1792 dipeptide transport system permease protein dppB
1793 ABC transporter, permease protein
1814 conserved hypothetical protein
1818 conserved hypothetical protein
1846 septum formation protein Maf
1847 putative rRNA large subunit m3Psi methyltransferase RlmH
1848 iojap-like ribosome-associated protein
1849 nicotinate-nucleotide adenylyltransferase
1850 coproporphyrinogen III oxidase, aerobic
1851 phosphoribosylamine--glycine ligase
1852 DNA-binding regulatory protein, YebC/PmpR family
1854 aminotransferase, class III
1871 Integral membrane protein domain protein
1873 quinone oxidoreductase
1876 PROBABLE THIOSULFATE SULFURTRANSFERASE PROTEIN
1887 ring hydroxylating alpha subunit, catalytic domain protein
1892 geranyltranstransferase PA4043
1894 putative GTP cyclohydrolase
1895 30s ribosomal protein s6.
1896 primosomal replication protein N
1897 ribosomal protein S18 (rpsR)
1898 ribosomal protein L9
1900 replicative DNA helicase
1902 4-hydroxythreonine-4-phosphate dehydrogenase
1903 PhoH family protein
1904 bacterioferritin comigratory protein (AHPC/TSA family)
1905 membrane protein
1906 aminotransferase
1907 homoserine dehydrogenase
1908 threonine synthase
1910 fructose-bisphosphatase
1911 aminopeptidase N PA3083
1919 glycolate oxidase subunit GlcD
1920 glycolate oxidase subunits GlcE and GlcF
1931 tRNA (5-methylaminomethyl-2-thiouridylate)-methyltransferase
1932 sulfite exporter TauE/SafE
1933 glutathione S-transferase
1934 adenylosuccinate lyase
1935 cytochrome C'
1936 prokaryotic cytochrome b561
1938 Domain of unknown function, putative
1955 homocysteine synthase PA5025
1957 dna-binding protein hu-beta
1958 TonB protein, C-terminal domain protein
1961 regulatory protein homolog
1962 GHKL domain protein
1963 putative potassium uptake protein
1964 argininosuccinate synthase
1965 ornithine carbamoyltransferase
1969 4-phosphoerythronate dehydrogenase
1977 ribosomal protein S20

1980 KR domain protein
1981 adenylate kinase
1982 3-deoxy-D-manno-octulosonate cytidylyltransferase
1983 Protein of unknown function (DUF343) superfamily
1985 transporter, MotA/TolQ/ExbB proton channel family protein
1989 beta-ketoadipyl coa thiolase
1990 conserved hypothetical protein
1992 ATP-dependent Clp protease adaptor protein ClpS
1994 bifunctional penicillin binding protein 1C precursor
1995 ATP-dependent clp protease ATP-binding
1997 hypothetical protein
2124 transcriptional regulator, LuxR family domain protein
2125 threonyl-tRNA synthetase
2126 translation initiation factor IF-3
2127 glutathione synthase
2128 PTS system, IIA component
2129 phosphocarrier protein HPr
2130 phosphoenolpyruvate-protein phosphotransferase
2132 ornithine cyclodeaminase
2133 Receptor family ligand binding region superfamily
2144 signal peptidase II
2145 isoleucyl-tRNA synthetase
2147 phosphoribosylglycinamide formyltransferase
2150 Fatty acid desaturase domain protein
2151 putative DNA helicase II
2154 hypothetical adenine-specific methylase nma1912
2155 succinyl-diaminopimelate desuccinylase
2247 lysine--tRNA ligase
2251 single-stranded-DNA-specific exonuclease RecJ
2252 lipoprotein, releasing system, transmembrane protein, LolC/E family
2253 lipoprotein releasing system atp-binding protein lold.
2456 argininosuccinate lyase
2458 NAD+ synthetase
2474 Chorismate lyase superfamily
2475 pseudouridylate synthase
2480 protein
2482 peptidase family M3
2484 fold bifunctional protein [includes: methylenetetrahydrofolatedehydrogenase
2485 transcriptional regulatory protein fixj.
2486 tw-component sensor kinase
2487 pyruvate dehydrogenase (acetyl-transferring), homodimeric type
2490 major flagellin precursor
2491 rna polymerase sigma factor for flagellar operon
2494 chemotaxis protein MotA
2527 flagellar biosynthetic protein FliQ
2528 flagellar biosynthetic protein FliP
2529 flagellar biosynthetic protein flip.
2532 flagellar flil protein.
2543 FliT , putative
2544 flagellar protein FliS
2545 Flagellar hook-associated protein 2
2547 bifunctional protein FolC

2548 HYPOTHETICAL TRANSMEMBRANE PROTEIN
2549 CvpA family protein
2550 amidophosphoribosyltransferase
2553 protein-P-II uridylyltransferase
2554 methionine aminopeptidase, type I
2555 ribosomal protein S2
2556 translation elongation factor Ts
2557 UMP kinase
2558 ribosome recycling factor
2559 undecaprenyl diphosphate synthase VC2256
2561 1-deoxy-D-xylulose 5-phosphate reductoisomerase
2563 outer membrane protein assembly complex, YaeT protein
2564 Outer membrane protein (OmpH-like) superfamily
2565 UDP-3-O-[3-hydroxymyristoyl] glucosamine N-acyltransferase
2566 beta-hydroxyacyl-(acyl-carrier-protein) dehydratase FabZ
2567 acyl-[acyl-carrier-protein]--UDP-N-acetylglucosamine O-acyltransferase
2568 lipid-A-disaccharide synthase
2569 ribonuclease HII
2570 PUTATIVE TRNA/RRNA METHYLTRANSFERASE PROTEIN
2571 kinase/pyrophosphorylase
2572 pyruvate, water dikinase
2578 Aromatic-Rich Protein Family superfamily
2579 protein yfjf
2581 enoyl-CoA hydratase/isomerase family protein
2584 Integral membrane protein superfamily
2585 glycine cleavage T-protein
2587 dTMP kinase
2588 DNA polymerase III, delta' subunit
2822 DoxD-like family superfamily
2827 conserved hypothetical protein
2830 glutathione peroxidase
2835 lipoprotein, putative
2838 D-methionine-binding lipoprotein MetQ
2847 ABC transporter, ATP-binding protein
2848 high-affinity branched-chain amino acid transport, ATP-binding protein
2849 branched-chain amino acid ABC transporter, permease protein
2850 high-affinity branched-chain amino acid ABC transporter, permease protein LivH
2851 leucine-, isoleucine-, valine-, threonine-, and alanine-bindingprotein precursor
2854 ABC transporter, solute-binding protein
2857 sn-glycerol-3-phosphate transport atp-binding protein ugpc.
2858 leucine-, isoleucine-, valine-, threonine-, and alanine-bindingprotein precursor
2884 reactive intermediate/imine deaminase
2889 ferredoxin--nadp reductase
2891 ABC transporter, permease protein
2894 pirin family protein
2896 nitrogen regulation protein NR(I)
2898 glutamine synthetase, type I
2899 oxygen-independent coproporphyrinogen III oxidase, putative
2900 non-canonical purine NTP pyrophosphatase RdgB
2911 NlpC/P60 family protein
2915 guanylate kinase
2916 DNA-directed RNA polymerase, omega subunit

2917 gtp pyrophosphokinase
2953 GDSL-like protein
2954 PPIC-type PPIASE domain protein
2956 protein hi0441
2958 glutaredoxin domain protein
2959 heavy metal-associated domain protein
2960 Cu(I)-responsive transcriptional regulator
2963 putative exported protein
2964 conserved hypothetical protein
2968 acyl-phosphate glycerol 3-phosphate acyltransferase
2970 Xanthine/uracil permeases family subfamily
2972 thioredoxin 2
2974 ABC transporter, permease protein
2976 ABC transporter, ATP-binding protein
2977 putative ABC transporter, ATP-binding protein YbiT
2984 muramoyltetrapeptide carboxypeptidase
2985 cytidine and deoxycytidylate deaminase family protein
2986 conserved hypothetical protein
2988 potassium efflux system protein phag
2989 pH adaptation potassium efflux system protein F
2990 putative pH adaptation potassium efflux protein
2993 putative lipoprotein
3000 hypothetical protein
3039 transcriptional regulator
3040 nitric oxide dioxygenase
3041 uncharacterized conserved protein, YqeY
3042 dna polymerase iv
3043 conserved domain protein
3045 peptidase
3046 penicillin-binding protein, 1A family
3108 RNA polymerase sigma factor RpoD
3110 ribosomal protein S21
3111 Phosphoribosyl transferase domain, putative
3112 adenylosuccinate synthase
3113 atp phosphoribosyltransferase regulatory subunit.
3114 HflC-like protein
3115 HflK protein
3116 GTP-binding protein HflX
3117 hfq protein
3118 histidinol-phosphate transaminase
3119 ribosome-associated GTPase EngA
3120 outer membrane assembly lipoprotein YfgL
3121 conserved hypothetical protein
3122 histidine--tRNA ligase
3123 4-hydroxy-3-methylbut-2-en-1-yl diphosphate synthase
3124 DNA-binding helix-turn-helix protein
3125 23S rRNA m2A2503 methyltransferase
3126 nucleoside diphosphate kinase
3133 tRNA (guanine-N1)-methyltransferase
3134 putative 16S rRNA processing protein RimM
3159 FAD dependent oxidoreductase
3160 putative CDP-6-deoxy-L-threo-D-glycero-4-hexulose-3-dehydrase reductase

3162 MOSC N-terminal beta barrel domain protein
3169 diaminobutyrate acetyltransferase
3177 DNA topoisomerase IV, A subunit
3179 DNA gyrase, B subunit, C-terminal domain protein
3181 thioredoxin
3182 transcription termination factor Rho
3184 conserved hypothetical protein
3187 carboxymethylenebutenolidase family superfamily
3188 metal cation transporter, ZIP domain protein
3194 ribosome-binding factor A
3197 conserved hypothetical protein
3199 segregation and condensation protein B
3204 maleylacetoacetate isomerase
3205 4-hydroxy-3-methylbut-2-enyl diphosphate reductase
3206 peptidyl-prolyl cis-trans isomerase, FKBP-type
3207 dna repair protein radc homolog.
3208 arylformamidase
3209 tryptophan 2,3-dioxygenase
3210 ribosomal protein L31
3212 MATE efflux family protein
3214 DNA repair protein RadA
3219 alanine racemase
3244 NUDIX domain protein
3288 queuine synthase
3291 CDP-diacylglycerol--glycerol-3-phosphate 3-phosphatidyltransferase
3292 excinuclease ABC, C subunit
3293 beta-hexosaminidase
3294 holo-[acyl-carrier-protein] synthase
3295 pyridoxal phosphate biosynthetic protein PdxJ
3297 multiple transferable resistance system protein MtrD
3301 putative lipoprotein
3303 sulfate/thiosulfate import atp-binding protein cysa
3304 ABC transporter integral membrane protein
3305 Protein of unknown function (DUF534) superfamily
3313 acetyl-CoA C-acyltransferase
3315 glycine betaine transport atp-binding protein opuaa
3316 inner membrane protein (AF111939)
3317 ABC transporter, quaternary amine uptake transporter family, substrate-binding protein
3319 drug resistance transporter, Bcr/CflA family
3321 ribosomal protein L33
3326 leucine--tRNA ligase
3327 LPS export system outer membrane protein LptE
3328 DNA polymerase III, delta subunit
3329 glutamate-5-semialdehyde dehydrogenase
3360 dna-3-methyladenine glycosylase
3361 ArsC family subfamily
3362 putrescine transport system permease protein poti.
3363 putrescine transport system permease protein poth.
3364 polyamine ABC transporter, ATP-binding protein
3365 ABC transporter, solute-binding protein
3366 lipopolysaccharide kinase, Kdo/WaaP family
3368 permease, putative domain protein

3370 Tripartite ATP-independent periplasmic transporters, DctQ component family
3376 phenylacetic acid degradation protein paai
3377 probable enoyl-coa hydratase paag
3378 phenylacetic acid degradation protein paaN
3380 phenylacetic acid degradation protein paac
3381 phenylacetic acid degradation protein paab
3383 phenylacetic acid degradation operon negative regulatory protein paax
3386 oligoribonuclease
3387 peptidase, M48 family
3388 GTPase YjeQ
3391 ribosomal protein L19
3402 AhpC/TSA family protein
3403 Acyl-CoA dehydrogenase, C-terminal domain protein
3406 histone deacetylase family protein
3407 membrane-bound lytic murein transglycosylase b precursor
3410 ADP-L-glycero-D-manno-heptose-6-epimerase
3411 bifunctional protein RfaE, domain I
3412 PUTATIVE TRANSMEMBRANE PROTEIN
3413 conserved hypothetical protein
3414 integration host factor beta-subunit
3415 ribosomal protein S1
3416 cytidylate kinase
3417 3-phosphoshikimate 1-carboxyvinyltransferase
3419 chorismate mutase/prephenate dehydratase NMB0446
3420 phosphoserine aminotransferase
3421 DNA gyrase, A subunit
3422 outer membrane protein A
3423 3-demethylubiquinone-9 3-O-methyltransferase
3424 phosphoglycolate phosphatase
3502 lytic murein transglycosylase
3503 PF04305 family protein
3505 PF08909 domain protein
3509 CsbD-like family
3511 osmotically inducible protein Y
3513 4Fe-4S binding domain protein
3515 iron permease FTR1 family protein
3516 cupredoxin-like domain protein
3517 Fe2+ transport protein
3518 phbF protein (AF026544)
3519 acetoacetyl-coa reductase
3520 poly-beta-hydroxybutyrate polymerase
3523 outer membrane assembly lipoprotein YfiO
3524 probable enzyme Z2851
3578 dihydrolipoyl dehydrogenase
3580 2-oxoglutarate dehydrogenase, E1 component
3583 pyruvate dehydrogenase e1 component
3587 citrate synthase I
3589 succinate dehydrogenase (EC 1.3.99.1)
3591 succinate dehydrogenase, hydrophobic membrane anchor protein
3592 succinate dehydrogenase, cytochrome b556 chain
3593 UbiC transcription regulator-associated domain protein
3594 malate dehydrogenase

3600 Uncharacterised protein family (UPF0093) superfamily
3602 hypothetical protein
3606 transferase hexapeptide repeat protein
3607 chaperonin HslO
3613 cell division protein ftsb homolog
3617 CTP synthase
3618 metallopeptidase family M24
3620 peptidase family T4
3621 dipeptide transport system permease protein dppc
3622 dipeptide transport system permease protein
3625 L-asparaginase
3626 SIS domain protein
3629 thioesterase family protein
3630 electron-transferring-flavoprotein dehydrogenase
3631 NAD(P)H-binding protein, PF13460 family
3640 NlpC/P60 family protein
3642 transcription regulator CysB PA1754
3644 ABC transporter substrate-binding protein
3646 LPS export ABC transporter permease LptF
3647 cytosol aminopeptidase family, catalytic domain protein
3648 DNA polymerase III, chi subunit
3650 Uncharacterized protein family UPF0005, putative
3655 DNA repair protein RecO
3656 GTP-binding protein Era
3657 ribonuclease iii
3659 GTP-binding protein LepA
3660 putative serine protease MucD
3661 PUTATIVE SIGMA-E FACTOR REGULATORY (NEGATIVE REGULATOR) PROTEIN
3662 Anti sigma-E protein RseA, N-terminal domain family
3663 rpoE protein homolog
3665 beta-ketoacyl synthase
3666 acyl carrier protein
3667 3-oxoacyl-(acyl-carrier-protein) reductase
3668 [acyl-carrier-protein] S-malonyltransferase
3669 beta-ketoacyl-acyl-carrier-protein synthase III
3670 fatty acid/phospholipid synthesis protein PlsX
3671 ribosomal protein L32
3672 hypothetical protein
3673 maf protein
3674 Tetrapyrrole (Corrin/Porphyrin) Methylases, putative
3675 putative exported hydrolase
3676 ferric reductase-like transmembrane component
3679 ribosomal large chain pseudouridine synthase C
3685 tryptophan synthase, alpha subunit
3686 tryptophan synthase, beta subunit
3690 flavin reductase domain protein
3692 nicotinate phosphoribosyltransferase
3695 intracellular poly[D(-)-3-hydroxybutyrate] depolymerase
3697 endonuclease III
3700 Receptor family ligand binding region superfamily
3707 Branched-chain amino acid transport system / permease component superfamily
3708 Receptor family ligand binding region superfamily

3712 PilB-related protein
3713 intracellular septation protein A
3714 PPIC-type PPIASE domain protein
3717 chromosome segregation protein SMC
3718 transporter, major facilitator family protein
3720 tRNA threonylcarbamoyl adenosine modification protein YeaZ
3723 alkyl hydroperoxide reductase
3739 conserved hypothetical protein
3741 NADH dehydrogenase I, N chain NMB0259
3742 proton-translocating NADH-quinone oxidoreductase, chain M
3743 nadh-quinone oxidoreductase chain l
3744 NADH dehydrogenase I, J chain NMB0253
3745 NADH2 dehydrogenase (ubiquinone) (EC 1.6.5.3) I chain I
3747 NADH dehydrogenase
3749 NADH-quinone oxidoreductase, F subunit
3750 NADH dehydrogenase I, E chain NMB0245
3751 NADH dehydrogenase I, D subunit
3752 respiratory-chain NADH dehydrogenase, 30 kDa subunit
3754 NADH2 dehydrogenase (ubiquinone) (EC 1.6.5.3) I chain A
3755 outer membrane porin protein bp0840 precursor
3757 HYPOTHETICAL TRANSMEMBRANE PROTEIN
3759 PAP2 homolog protein
3760 calcineurin-like phosphoesterase family protein
3792 hypothetical protein
3793 threonine ammonia-lyase
3795 polyribonucleotide nucleotidyltransferase
3796 ribosomal protein S15
3797 lipoprotein, putative
3798 phosphatidylserine synthase PA4693
3799 ketol-acid reductoisomerase
3800 acetolactate synthase, small subunit
3801 acetolactate synthase, large subunit, biosynthetic type
3806 d-beta-hydroxybutyrate dehydrogenase
3819 hypothetical protein
3822 seryl-tRNA synthetase
3823 MgsA AAA+ ATPase family protein
3825 dna translocase ftsk
3826 thioredoxin-disulfide reductase
3837 isocitrate dehydrogenase, NADP-dependent
3838 conserved hypothetical protein
3841 ATPase, AAA family
3842 hypothetical protein
3846 chaperone protein dnaj
3847 chaperone protein DnaK
3849 hypothetical protein
3850 co-chaperone GrpE
3851 conserved hypothetical protein
3852 ferrochelatase
3853 heat-inducible transcription repressor HrcA
3858 dihydrodipicolinate reductase
3884 PF11161 family protein
3887 NUDIX domain protein

3890 dihydroorotate dehydrogenase (fumarate)
3892 Uncharacterized protein family, UPF0065 superfamily
3894 transcriptional regulator, IclR family family
3895 Probable molybdopterin binding domain protein
3896 hypothetical protein
3897 thioredoxin domain protein
3898 DEAD/DEAH box helicase
3899 AzlC protein
3902 conserved hypothetical protein
3903 Esterase/lipase/thioesterase family active site
3904 peptidase, M48 family
3905 UTP-glucose-1-phosphate uridylyltransferase
3910 aminopeptidase P PA5224
3913 TIM-barrel protein, nifR3 family
3916 crossover junction endodeoxyribonuclease RuvC
3921 ATP-dependent DNA helicase RecQ
3923 transcription regulatory protein ompR
3924 ATPase, histidine kinase-, DNA gyrase B-, and HSP90-like domain protein protein
3927 glutaminyl-tRNA synthetase
3931 osmotically inducible lipoprotein B precursor
3932 isochorismatase family protein superfamily
3946 conserved hypothetical protein
3950 PUTATIVE PARA-AMINOBENZOATE SYNTHETASE COMPONENT I PROTEIN
3958 pyridoxamine 5'-phosphate oxidase
3959 flavin reductase-like protein
3960 FAD dependent oxidoreductase
3961 formyltetrahydrofolate deformylase
3968 tripartite tricarboxylate transporter family receptor
3970 fumarate hydratase, class II
3972 N-acetylmuramoyl-L-alanine amidase
3975 tRNA delta(2)-isopentenylpyrophosphate transferase
3976 phosphoribosylformylglycinamidine cyclo-ligase
3977 DnaA regulatory inactivator Hda
3978 poly(A) polymerase
3979 2-amino-4-hydroxy-6-hydroxymethyldihydropteridine diphosphokinase
4004 glycine--tRNA ligase, alpha subunit
4005 glycine--tRNA ligase, beta subunit
4006 haloacid dehalogenase-like hydrolase, putative
4007 probable acyltransferase PA0005
4009 lactoylglutathione lyase
4010 polypeptide deformylase
4013 transporter gate domain protein
4015 glycerate kinase
4016 dimethyladenosine transferase
4019 phosphotransferase enzyme family protein
4020 MobA-like NTP transferase domain protein
4021 putative inner membrane protein
4080 ABC transporter substrate binding protein
4081 d-methionine transport system permease protein meti
4082 d-methionine transport atp-binding protein metn
4083 glycoside hydrolase, family 31
4088 putative hydrolase

4090 putative membrane protein
4096 transcription regulatory protein, asnc family
4101 conserved hypothetical protein
4102 preprotein translocase, SecA subunit
4103 peptidase, M23 family
4104 flagellar hook-length control protein FliK
4105 UDP-3-O-[3-hydroxymyristoyl] N-acetylglucosamine deacetylase
4106 cell division protein FtsZ
4107 cell division protein FtsA
4108 cell division protein FtsQ
4109 D-alanine--D-alanine ligase
4110 UDP-N-acetylmuramate--alanine ligase
4111 undecaprenyldiphospho-muramoylpentapeptide beta-N-acetylglucosaminyltransferase
4112 cell division protein NMA2063
4113 UDP-N-acetylmuramoyl-L-alanine--D-glutamate ligase
4114 phospho-N-acetylmuramoyl-pentapeptide-transferase
4115 UDP-N-acetylmuramoyl-L-alanyl-D-glutamate--2,6-diaminopimelate ligase
4116 penicillin-binding protein, transpeptidase domain protein
4117 cell division protein FtsL
4118 S-adenosyl-methyltransferase MraW
4119 protein MraZ
4121 dihydroorotase, homodimeric type
4122 hippurate hydrolase
4123 swib (ym74) complex protein
4125 winged helix-turn-helix DNA-binding protein
4126 4-hydroxyphenylpyruvate dioxygenase
4128 cytochrome c-552 precursor
4130 prolipoprotein diacylglyceryl transferase
4138 LysR substrate-binding domain protein
4143 MutT/nudix family protein VC0671
4144 prolyl-tRNA synthetase
4147 TolR protein XF1899
4150 tolb protein precursor
4151 peptidoglycan-associated lipoprotein
4152 conserved hypothetical protein
4174 universal stress protein family
4175 conserved hypothetical protein TIGR01244
4176 transcriptional regulator, gntR family domain protein
4190 DNA polymerase III, epsilon subunit
4193 ribonuclease HI
4194 methyltransferase domain protein
4195 hydroxyacylglutathione hydrolase XF2160
4196 LysM domain protein
4197 enoyl-[acyl-carrier-protein] reductase [nadh]
4202 hypothetical protein
4255 riboflavin biosynthesis protein RibD
4258 transcriptional regulator, NrdR family
4259 glycine hydroxymethyltransferase
4260 conserved hypothetical protein TIGR00481
4261 tyrosyl-tRNA synthetase
4263 Uncharacterised protein family (UPF0075) superfamily
4264 hesB family protein VC0627

4266 N-acetyl-gamma-glutamyl-phosphate reductase
4267 ribosomal protein S9
4268 ribosomal protein L13
4271 TldD/PmbA family protein
4272 PF04751 family protein
4273 carboxylesterase XF1624
4282 succinyl-CoA ligase-like flavodoxin domain protein
4283 glutamate--tRNA ligase
4288 ribonucleoside-diphosphate reductase, alpha subunit
4289 ribonucleoside-diphosphate reductase beta chain
4291 YGGT family domain protein
4298 carbohydrate kinase, PfkB family
4299 possible membrane protein
4301 acetyl-CoA carboxylase, biotin carboxylase
4308 TonB protein XF1957
4311 LPS ABC transporter, permease protein LptG
4312 bis(5'-nucleosyl)-tetraphosphatase (symmetrical)
4313 acyltransferase
4316 RNAse H domain protein, YqgF family
4319 thiamine-phosphate diphosphorylase
4320 glutamate-1-semialdehyde-2,1-aminomutase
4322 transcription regulator aldR
4323 conserved hypothetical protein
4324 AMP-binding enzyme domain protein
4325 acyl-coa dehydrogenase, short-chain specific, mitochondrial
4326 enoyl-CoA hydratase/isomerase family protein
4329 high-affinity branched-chain amino acid transport atp-binding proteinlivf
4335 probable transcription regulator, putative
4337 arginine biosynthesis bifunctional protein ArgJ
4339 putative thiamine-phosphate diphosphorylase
4340 efflux transporter, outer membrane factor lipoprotein, NodT family
4343 efflux transporter, RND family, MFP subunit
4344 PF07072 family protein
4345 dephospho-CoA kinase
4346 type IV prepilin leader peptidase
4347 conserved hypothetical protein
4348 pantoate--beta-alanine ligase
4349 segregation and condensation protein A
4350 PF11943 family protein
4351 drug resistance MFS transporter, drug:H+ antiporter-2 family
4352 glycerol kinase
4354 Uncharacterized protein family, UPF0065 superfamily
4355 conserved hypothetical protein
4356 amino acid ABC transporter, permease protein
4357 amino acid ABC transporter, permease protein
4358 general L-amino acid-binding periplasmic protein AapJ
4360 cell division protein FtsX homolog
4361 chemotaxis protein CheD
4363 A/G-specific adenine glycosylase
4366 methionine synthase
4369 thiazole biosynthesis protein ThiG
4370 putative phosphomethylpyrimidine kinase

4371 protein-l-isoaspartate o-methyltransferase
4374 aut protein
4379 ubiquinone/menaquinone biosynthesis methlytransferase UbiE
4380 phosphate regulon transcriptional regulatory protein PhoB
4381 phosphate regulon sensor kinase PhoR
4385 phosphoglycerate dehydrogenase
4387 glyoxalase family protein superfamily
4388 sensor histidine kinase inhibitor, KipI family
4389 allophanate hydrolase subunit 2
4390 lactam utilization protein ycsF
4398 PF12447 family protein
4400 threonine dehydratase, biosynthetic
4404 adenine phosphoribosyltransferase
4406 thiol reductant ABC exporter, CydD subunit
4407 putative exported protein
4408 cytochrome d ubiquinol oxidase, subunit II
4409 cytochrome d oxidase chain (AF284438) AGR_L_1529
4411 phosphoribosylglycinamide formyltransferase 2
4412 polysialic acid capsule expression protein XF1413
4413 3-deoxy-d-manno-octulosonate 8-phosphate phosphatase
4414 lipopolysaccharide transport periplasmic protein LptA
4415 LPS export ABC transporter ATP-binding protein
4416 putative PTS IIA-like nitrogen-regulatory protein PtsN
4420 Uncharacterised P-loop ATPase protein family (UPF0042) superfamily
4421 lipoprotein A-like protein
4426 HYPOTHETICAL SIGNAL PEPTIDE PROTEIN
4427 redoxin
4428 outer membrane protein XF0873
4429 acetylglutamate kinase
4430 hydrolase, probable NMB0040
4431 transcriptional regulator, TetR family
4435 rod shape-determining protein roda
4436 penicillin-binding protein 2
4437 conserved hypothetical protein
4438 rod shape-determining protein MreC
4439 regulator of ftsI
4441 aspartyl/glutamyl-tRNA(Asn/Gln) amidotransferase, A subunit
4462 muropeptide transporter
4495 transcriptional regulatory protein
4496 nitronate monooxygenase
4498 enoyl-CoA hydratase/isomerase family protein family
4499 YjeF family C-terminal domain protein
4500 hypothetical protein
4507 hypothetical protein
4511 uroporphyrinogen decarboxylase
4512 transcriptional regulator, Fis domain protein
4513 ATP synthase F1, epsilon subunit
4514 ATP synthase F1, beta subunit
4515 ATP synthase F1, gamma subunit
4516 ATP synthase F1, alpha subunit
4517 ATP synthase F1, delta subunit
4518 ATP synthase F0, B subunit

4520 ATP synthase F0, A subunit
4522 enoyl-CoA hydratase/isomerase family protein
4523 peptidase, M61 glycyl aminopeptidase family protein
4524 pyridine nucleotide-disulfide oxidoreductase
4526 ATPases of the PP superfamily BMEI1132
4528 conserved hypothetical protein
4529 protein visc
4530 MltA family family
4531 apaG protein
4533 phosphoglycolate phosphatase
4534 anthranilate synthase component I
4726 glycosyltransferase, group 1 family protein
4727 nucleotide sugar dehydrogenase
4728 putative membrane protein
4729 UDP-N-acetylglucosamine diphosphorylase/glucosamine-1-phosphate N-acetyltransferase
4731 PF12512 family protein
4732 SCO1/2 family protein
4733 protoheme IX farnesyltransferase
4734 cytochrome oxidase assembly protein
4742 NUDIX domain protein
4744 calcineurin-like phosphoesterase family protein
4745 transcription initiation factor sigma 32
4746 hypothetical protein
4747 S-formylglutathione hydrolase
4749 hypothetical protein
4750 glutamate synthase (NADH2) (EC 1.4.1.14) large chain
4751 pyridine nucleotide-disulfide oxidoreductase
4755 toluene tolerance protein XF0420
4756 outer membrane lipid asymmetry maintenance protein MlaD
4757 lipid assymetry maintenance protein MlaA
4758 Toluene tolerance, Ttg2 superfamily
4761 BolA/YrbA family protein NMB0009
4762 UDP-N-acetylglucosamine 1-carboxyvinyltransferase
4763 ATP phosphoribosyltransferase
4764 histidinol dehydrogenase
4766 imidazoleglycerol-phosphate dehydratase
4767 imidazole glycerol phosphate synthase, glutamine amidotransferase subunit-like protein
4768 1-(5-phosphoribosyl)-5-[(5-phosphoribosylamino)methylideneamino]imidazole-4-carboxamide isomerase
4769 imidazoleglycerol phosphate synthase, cyclase subunit
4770 phosphoribosyl-ATP diphosphatase
4771 HIT family hydrolase
4772 sec-independent protein translocase protein tata/e homolog
4774 Sec-independent protein translocase TatC
4777 conserved hypothetical protein TIGR00486
4778 large conductance mechanosensitive channel protein
4779 ubiquinol-cytochrome c reductase, iron-sulfur subunit
4780 cytochrome B/b6/PetD
4781 cytochrome c1 family protein
4782 stringent starvation protein A
4783 stringent starvation protein B XF0912
4815 conserved hypothetical protein
4822 DEAD/DEAH box helicase

4823 homoserine/homoserine lactone efflux protein
4824 peptide chain release factor 3
4825 hypothetical protein
4845 hypothetical protein
4846 hypothetical protein
4847 arginine--tRNA ligase
4848 sporulation and cell division repeat protein
4849 thiol:disulfide interchange protein dsba precursor
4850 cystathionine beta-lyase
4852 metallo-beta-lactamase superfamily domain protein
4856 Probable thiolase
4857 carboxyl transferase domain protein
4860 transaminase, acetylornithine/succinylornithine family
4862 alanine racemase, N-terminal domain protein
4866 PF04361 family protein
4874 putative gluconate 5-dehydrogenase
4875 PROBABLE ALDEHYDE DEHYDROGENASE OXIDOREDUCTASE PROTEIN
4897 DNA gyrase, B subunit
4900 chromosomal replication initiator protein DnaA
4901 ribosomal protein L34

Supplementary References

s1. Parkhill J, Sebaihia M, Preston A, Murphy LD, Thomson N, Harris DE, Holden MT, Churcher CM, Bentley SD, Mungall KL, Cerdeno-Tarraga AM, Temple L, James K, Harris B, Quail MA, Achtman M, Atkin R, Baker S, Basham D, Bason N, Cherevach I, Chillingworth T, Collins M, Cronin A, Davis P, Doggett J, Feltwell T, Goble A, Hamlin N, Hauser H, Holroyd S, Jagels K, Leather S, Moule S, Norberczak H, O'Neil S, Ormond D, Price C, Rabbinowitsch E, Rutter S, Sanders M, Saunders D, Seeger K, Sharp S, Simmonds M, Skelton J, Squares R, Squares S, Stevens K, Unwin L, Whitehead S, Barrell BG, Maskell DJ (2003) Comparative analysis of the genome sequences of *Bordetella pertussis*, *Bordetella parapertussis* and *Bordetella bronchiseptica*. Nat Genet 35: 32-40.

s2. Register KB, Ivanov YV, Jacobs N, Meyer JA, Goodfield LL, Muse SJ, Smallridge WE, Brinkac L, Kim M, Sanka R, Harvill ET, Losada L (2015) Draft Genome Sequences of 53 Genetically Distinct Isolates of *Bordetella bronchiseptica* Representing 11 Terrestrial and Aquatic Hosts. Genome Announc 3: e00152-15.

s3. Nicholson TL, Shore SM, Bayles DO, Register KB, Kingsley RA (2014) Draft Genome Sequence of the *Bordetella bronchiseptica* Swine Isolate KM22. Genome Announc 2: e00670-14.

s4. Park J, Zhang Y, Buboltz AM, Zhang X, Schuster SC, Ahuja U, Liu M, Miller JF, Sebaihia M, Bentley SD, Parkhill J, Harvill ET (2012) Comparative genomics of the classical *Bordetella* subspecies: the evolution and exchange of virulence-associated diversity amongst closely related pathogens. BMC Genomics 13: 545.

s5. Register KB, Ivanov YV, Harvill ET, Davison N, Foster G (2015) Novel, host-restricted genotypes of *Bordetella bronchiseptica* associated with phocine respiratory tract isolates. Microbiology 161: 580-592.

s6. Zhang S, Xu Y, Zhou Z, Wang S, Yang R, Wang J, Wang L (2011) Complete genome sequence of *Bordetella pertussis* CS, a Chinese pertussis vaccine strain. J Bacteriol 193: 4017-4018.

s7. Harvill ET, Goodfield LL, Ivanov Y, Meyer JA, Newth C, Cassiday P, Tondella ML, Liao P, Zimmerman J, Meert K, Wessel D, Berger J, Dean JM, Holubkov R, Burr J, Liu T, Brinkac L, Kim M, Losada L (2013) Genome Sequences of 28 *Bordetella pertussis* U.S. Outbreak Strains Dating from 2010 to 2012. Genome Announc 1: e01075-13.

s8. Harvill ET, Goodfield LL, Ivanov Y, Smallridge WE, Meyer JA, Cassiday PK, Tondella ML, Brinkac L, Sanka R, Kim M, Losada L (2014) Genome Sequences of Nine *Bordetella holmesii* Strains Isolated in the United States. Genome Announc 2: e00438-14.

s9. Tatti KM, Loparev VN, Ranganathanganakammal S, Changayil S, Frace M, Weil MR, Sammons S, Maccannell D, Mayer LW, Tondella ML (2013) Draft genome sequences of *Bordetella holmesii* strains from blood (F627) and nasopharynx (H558). Genome Announc 1: e00056-13.

s10. Register KB, Ivanov YV, Harvill ET, Brinkac L, Kim M, Losada L (2015) Draft Genome Sequences of Six *Bordetella hinzii* Isolates Acquired from Avian and Mammalian Hosts. Genome Announc 3: e00081-15.

s11. Shah NR, Moksa M, Novikov A, Perry MB, Hirst M, Caroff M, Fernandez RC (2013) Draft Genome Sequences of *Bordetella hinzii* and *Bordetella trematum*. Genome Announc 1: e00838-13.

s12. Sebaihia M, Preston A, Maskell DJ, Kuzmiak H, Connell TD, King ND, Orndorff PE, Miyamoto DM, Thomson NR, Harris D, Goble A, Lord A, Murphy L, Quail MA, Rutter S, Squares R, Squares S, Woodward J, Parkhill J, Temple LM (2006) Comparison of the genome sequence of the poultry pathogen *Bordetella avium* with those of *B. bronchiseptica*, *B. pertussis*, and *B. parapertussis* reveals extensive diversity in surface structures associated with host interaction. J Bacteriol 188: 6002-6015.

s13. Gross R, Guzman CA, Sebaihia M, dos Santos VA, Pieper DH, Koebnik R, Lechner M, Bartels D, Buhrmester J, Choudhuri JV, Ebensen T, Gaigalat L, Herrmann S, Khachane AN, Larisch C, Link S, Linke B, Meyer F, Mormann S, Nakunst D, Ruckert C, Schneiker-Bekel S, Schulze K, Vorholter FJ, Yevsa T, Engle JT, Goldman WE, Puhler A, Gobel UB, Goesmann A, Blocker H, Kaiser O, Martinez-Arias R (2008) The missing link: *Bordetella petrii* is endowed with both the metabolic versatility of environmental bacteria and virulence traits of pathogenic Bordetellae. BMC Genomics 9: 449.
